# Neuronal Properties in the Lateral Habenula and Adult–Newborn Interactions in Virgin Female and Male Mice

**DOI:** 10.1523/ENEURO.0414-24.2025

**Published:** 2025-02-11

**Authors:** Cheng-Hsi Wu, Manuel Mameli, Salvatore Lecca

**Affiliations:** ^1^Department of Fundamental Neuroscience, University of Lausanne, Lausanne 1003, Switzerland; ^2^Institut national de la santé et de la recherche médicale UMR-S 839, Paris 75005, France

**Keywords:** adult–newborn interactions, lateral habenula, neuronal excitability, pup aggression, pup retrieval, sex dimorphism

## Abstract

The behavioral interactions between adults and newborns are decisive for the fitness and the survival of offspring across the animal kingdom. In laboratory mice, while virgin females display caregiving behaviors, virgin males are rather neglectful or aggressive toward pups. Despite the importance of these behavioral variations, the underlying neural mechanisms remain poorly understood. Brain regions encoding these behaviors may exhibit sex-dependent functional differences at the baseline. Additionally, these structures might undergo sex-specific plasticity after adults interact with the offspring. Emerging evidence suggests sex-based differences in input connectivity, genetics, and receptor expression of the epithalamic lateral habenula (LHb). Moreover, LHb neuronal activity is instrumental for adult–newborn interactions. However, whether LHb neuronal function varies between sexes and/or undergoes adaptations following interactions with pups has not been fully investigated. In this study, we used in vivo and ex vivo single-cell electrophysiology to examine the basal LHb neuronal activity of virgin female and male mice. In a second set of experiments, we exposed mice to pups and recapitulated sex-based divergent behaviors. Recordings in acute slices showed no alterations in LHb firing properties, regardless of sex or pup exposure. These findings suggest that, although the LHb participates in adult behaviors toward pups, this is not mediated by sex-dependent functional differences or adaptations in the neuronal firing properties. Thus, this study provides new insights into the neural basis of sex-specific adult–newborn behaviors and the role of the LHb in these processes.

## Significance Statement

Previous studies highlight the importance of the lateral habenula (LHb) in guiding parental behaviors. Virgin female and male mice differ in their behavior toward pups: while females are parental, males neglect or attack pups. We investigated whether LHb neuronal activity or its plasticity after pup exposure presented sex differences in virgin mice. Our findings reveal that LHb activity does not differ between naive females and males, and it remains unchanged after pup exposure. This suggests a decoupling between sex-dimorphic behaviors toward newborns and activity of the LHb.

## Introduction

Sexual dimorphism in brain structure and function underlies sex differences in a variety of behaviors and pathological conditions across species (Giedd et al., 1997; Hernandez-Avila et al., 2004; de Vries and Södersten, 2009; Mohammadi et al., 2023). Social behaviors, for instance, exhibit significant sexual dimorphism (Bayless and Shah, 2016), and a notable example is adult–newborn interactions. Rodent studies demonstrate that females and males display distinct actions toward the offspring (Dulac et al., 2014). In adult laboratory mice, virgin females exhibit caregiving behaviors, similar to parenthood, while virgin males neglect or even show aggression toward pups (vom Saal, 1985; Stolzenberg and Rissman, 2011). This dimorphism in behavior may emerge from sexually differentiated functions in neuronal circuits responsible for adult–newborn interactions in mice (Kaplan et al., 2025). For instance, hormonal fluctuations produce sex-dependent changes in circuit connectivity and neuronal excitability, leading to sex-specific behaviors toward pups (Bosch and Neumann, 2012; Li et al., 2015; Zilkha et al., 2017; Froemke and Young, 2021; Inada et al., 2022; Smiley et al., 2022; Ammari et al., 2023).

Recent evidence suggests the lateral habenula (LHb) as a key candidate that can control sexually dimorphic behaviors, due to its sexual differences in anatomy, genetics, and receptor expression, as well as its control of adult–newborn interactions (Corodimas et al., 1992; Matthews-Felton et al., 1995; Lecca et al., 2023; Michel et al., 2024).

The LHb is a negative affect-encoding brain center that finely tunes the activity of neuromodulatory systems (dopamine and serotonin) to control aversive instinctive and learned behaviors (Mondoloni et al., 2022). Notably, the LHb expresses sex-dependent features. Rabies-assisted monosynaptic mapping of innervation onto the LHb reveals a sex-dependent difference between excitatory and inhibitory inputs weight (Liu et al., 2022). Single-cell transcriptomic analyses of LHb neuronal subpopulations demonstrate distinct genetic profiles between male and female mice (Lecca et al., 2023). Finally, immunochemistry studies indicate sex differences in the expression of estrogen receptors in the LHb, with females showing higher levels compared with males (Bell et al., 2023), while vasopressin fiber density is higher in males (Lonstein et al., 2005). Notably, both estrogen and vasopressin are hormones influencing sex-dependent social behaviors (Rosenblatt et al., 1998; Insel, 2010).

From a behavioral standpoint, the LHb guides adult–newborn interactions in virgin female rodents (Corodimas et al., 1992; Matthews-Felton et al., 1995; Lecca et al., 2023; Benedict et al., 2024). Indeed, a circuit-defined subpopulation within the LHb, targeted by the sexual dimorphic bed nucleus of the stria terminalis (BNST), integrates the aversive signals from a pup in distress (i.e., pup distress vocalization) and becomes excited during specific adult actions such as pup retrieval. LHb neuronal excitation is essential for this behavior (Lecca et al., 2023). Here, we tested the hypothesis that LHb firing properties can differ across sexes and may exhibit adaptation after pup exposure, thus reflecting potential sex-specific mechanisms underlying adult–newborn interactions.

To test this, we utilized in vivo and ex vivo single-cell recordings to investigate sex-based differences in LHb firing activity. Additionally, we exposed virgin females and males to a series of pups in an experimental arena to identify specific behavioral motifs characterizing sexual dimorphism in adult–newborn interactions. Finally, we assessed whether sex-divergent adult–newborn interactions lead to immediate sex-specific functional adaptations in LHb firing activity.

## Materials and Methods

### Experimental subjects

Experiments were performed on 8–11-week-old virgin C57BL/6J wild-type female and male mice (Janvier Labs). Mice were housed in groups of up to five, with water and food provided *ad libitum*, on a 12:12 h light/dark cycle (lights on at 7 A.M.) in individually ventilated cages (Innovive). Pups of Postnatal Day (P)2–6 were obtained from donor C57BL/6J pairs. All procedures were conducted in compliance with the Swiss National Institutional Guidelines on Animal Experimentation and were approved by the canton of Vaud Cantonal Veterinary Office Committee for Animal Experimentation (Switzerland; License VD3798).

### Adult–newborn interaction behavioral assessment

Unless stated differently (Extended Data Fig. 2-2), all mice were placed in a phenotyper box (Noldus, 58 cm H, 30 cm L, 30 cm W) for 30 min of habituation in group (maximum of five mice) a day before behavioral assessment. In addition to the top-view camera, an extra camera was placed on the side of the arena, ensuring clear video recording of the behavior from a lateral point of view. The behavioral arena was filled with standard wood chip bedding (Safe) and divided into two main zones: a nest zone in one corner with nesting material (paper Kleenex) and a pup zone in the opposite corner where the pup was introduced through an external sliding door. The video was recorded and controlled with EthoVision XT 15 (Noldus Information Technology).

On the second day, the experimental subject was placed again in the phenotyper to maximize familiarity with the environment. In the pup exposure group, following habituation, either one or a series of pups was introduced in the phenotyper, with each pup remaining for a maximum of 10 min. If a retrieval event occurred, another pup was immediately introduced. In the pup exposure group of male mice, once an attack event was observed, the experiment was terminated, and no further pup was introduced to the male mouse. The percentage of time spent pup grooming, nest building, and general locomotion was scored for each mouse tested. Females that retrieved at least four out of seven pups were considered “high-retrievers” (Extended Data Fig. 4-2). Males that attack pups were defined as “attackers” (Extended Data Fig. 4-4). One hour after the behavioral test, the mice were prepared for either in vivo or ex vivo electrophysiology recording. In total, mice went through 1.5 h isolated period in the phenotyper.

### In vivo single-unit electrophysiology

Mice were anaesthetized with isoflurane (Univentor; induction, 3.5%; maintenance, 2%) and placed in the stereotaxic apparatus (Kopf). Their body temperature was maintained at 36 ± 1°C using a feedback-controlled heating pad (CMA 450 Temperature Controller). The scalp was retracted, and a single burr hole was drilled above the LHb (AP, −1.3 to −1.6 mm; L, 0.4–0.65 mm; V, −2.4 to −3.0 mm) for the placement of a recording electrode. Electrical activity was recorded extracellularly with a glass microelectrode filled with 0.5 M sodium acetate and 2% pontamine sky blue (impedance 6–12 MΩ). The signal was filtered (bandpass 500–5,000 Hz), preamplified (DAM80, World Precision Instrument), amplified (NeuroLog System, Digitimer), and displayed on a digital storage oscilloscope (OX 530, Metrix).

Experiments were sampled on- and off-line by a computer connected to CED Power 1401 laboratory interface (Cambridge Electronic Design) running the Spike2 software (Cambridge Electronic Design).

At the end of the experiment, the electrode placement was determined with an iontophoretic deposit of pontamine sky blue dye (1.5 mA, continuous current for 200 s). Brains were rapidly removed and fixed in 4% paraformaldehyde solution. Recording positions were confirmed with pontamine sky blue dye on serial brain slices by vibratome sectioning (60 µm). Only recordings within the LHb were considered for further analysis.

The spontaneous activity of single units was recorded for ∼3 min. Within this study, we examined the electrophysiological properties of the spontaneous firing rate, coefficient of variation (standard deviation of interspike intervals/mean interspike interval; a measure of firing regularity) expressed as a percentage, a percentage of spikes in burst and the burst rate. Burst activity was defined by an interspike interval <20 ms and terminated when the interspike interval exceeded 60 ms, with a minimum of two spikes required to constitute a burst. The neurons without burst were not taken into account for burst rate analysis**.** For firing pattern analysis, autocorrelograms were generated using a 10 ms bin width for intervals up to 2 s, to qualitatively classify neurons as firing in the regular, irregular, or burst firing mode (Lecca et al., 2017; Bell et al., 2023). Autocorrelograms showing three or more regularly occurring peaks were characteristic of the regular firing pattern. An initial trough that rose smoothly to a steady state was classified as an irregular firing pattern, whereas an initial peak, followed by decay to a steady state, was indicating a burst pattern.

### Ex vivo patch-clamp electrophysiology

Animals were anesthetized with ketamine (150 mg/kg) and xylazine (10 mg/kg) intraperitoneal injections (Veterinary Office University of Lausanne). The brains were rapidly removed and placed into bubbled ice-cold solution (95% O_2_ and 5% CO_2_) containing the following (in mM): 110 choline Cl, 25 NaHCO3, 1.25 NaH2PO4, 2.5 KCl, 25 glucose, 11.6 ascorbic acid, 3.1 sodium pyruvate, 1.3 MgCl2, and 2.5 CaCl2. The brains were then sliced in the coronal plane at 250 µm with a vibratome (Campden Instruments).

Brain slices containing the LHb were incubated in artificial cerebrospinal fluid (ACSF) with the following reagents (in mM): 124 NaCl, 2.5 KCl, 26.2 NaHCO3, 1 NaH2PO4, 11 glucose, 7 MgCl2, and 0.5 CaCl2 at 34°C for 5 min. Subsequently, the brain slices were transferred to ACSF at room temperature and incubated for an hour before recording. The ACSF bath solution was maintained at 31°C with a flow rate of 2 ml/min.

Throughout the recordings, the electrical signal was filtered at 5 kHz and digitized at 10 kHz using MultiClamp 700B (Molecular Devices). Data acquisition was performed with the Clampex software. Glass electrodes (2–6 MΩ) were filled with a KGlu-based internal solution containing the following (in mM): 140 potassium gluconate, 5 KCl, 10 HEPES, 0.2 EGTA, 2 MgCl2, 4 Na2ATP, 0.3 Na3GTP, and 10 creatine phosphate, pH 7.3, ∼290 mOsm.

LHb neurons were first held in a voltage clamp at −50 mV, followed by current-clamp experiments. Neurons were defined as silent, active-burst, and active-nonburst based on their spiking activity. Active-burst cells were defined by the display of a burst event (a train of action potentials in rapid succession) during the 3 min baseline recording. The resting membrane potential (RMP) and the spontaneous firing activity were calculated for every cell patched. Cell capacitance and input resistance were recorded for each neuron. The input resistance was calculated dividing the voltage change and current injected in the hyperpolarization protocol. Voltage change was obtained as the differences between baseline and the steady state (last 100 ms at the hyperpolarization state). If an action potential fell in this period, input resistance would not be included in the analysis. The percentage of spikes in burst was reported for active neurons, and the burst rate was examined only for active-burst neurons. All the recordings were performed at the RMP of the neurons. Subsequently, different steps of positive current (ranging from +20 to +100 pA, in 20 pA increments, duration of 800 ms each) were injected to induce depolarization. The number of action potentials was analyzed within 800 ms depolarization protocol. A series of negative currents (ranging from −20 to −60 pA, in 20 pA increments, duration of 800 ms each) were injected to hyperpolarize neurons and induce rebound burst activity. The number of bursts were analyzed for 5 s after the end of the hyperpolarization protocol. For further identification of the recording position, we took pictures of every recording and visually separated the whole LHb into medial and lateral territory. We reconstructed the recording mapping based on the pictures, and for each mouse we recorded an even number of neurons within the two territories.

### Statistical analysis

Data are reported either as boxplots displaying the full range from minimum to maximum with all data points or as data plots representing mean ± SEM, connected by lines. Statistical analyses were performed using Prism (GraphPad). The statistical tests used in this study included the Student's *t* test, Mann–Whitney test, *χ*^2^ test, one-way ANOVA repeat measures followed by Tukey's multiple-comparison test, and two-way ANOVA repeat measures followed by Holm–Šídák post hoc test for the interaction between groups. A *p* < 0.05 was considered significant. Compiled data are expressed as mean ± SEM.

## Results

### LHb spontaneous activity in anesthetized female and male virgin mice

We first assessed LHb neuronal spontaneous activity in naive female and male mice using in vivo single-unit extracellular recordings under isoflurane anesthesia. Prior to the experiment, all mice were exposed to an experimental arena provided with nest material for a total of 30 min (see Materials and Methods). One hour after the exposure, we proceeded with the recordings.

**Figure 1.**
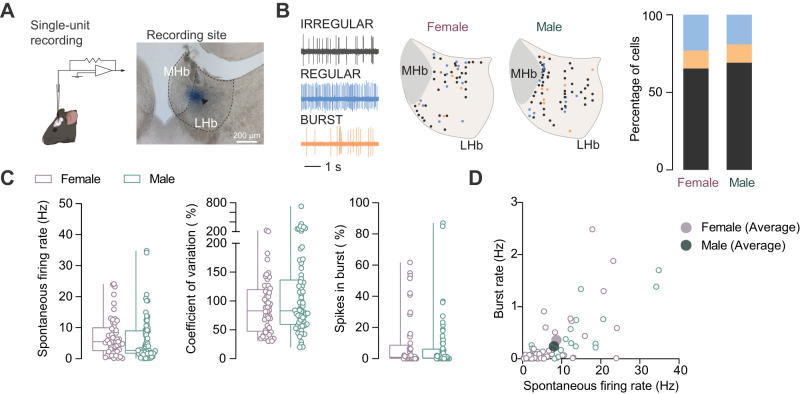
LHb spontaneous firing activity in naive females and males. ***A***, Schematics of recording and recording location example in LHb labeled with pontamine sky blue dye (MHb, medial habenula). ***B***, Sample traces showing the different firing pattern of LHb neurons labeled in different colors. Naive females, *N* = 6 mice. Naive males, *N* = 6 mice. Approximate location of each recorded neurons in naive female and male mice, color coded accordingly to the different firing patterns. Neurons recorded in the same location are labeled in gradient. The percentage of each firing pattern in females and males showed in different colors. Naive females, *n* = 52 cells. Naive males, *n* = 68 cells. ***C***, Boxplots with scatter points of the spontaneous firing rate (naive females vs naive males; 7.242 ± 0.867 Hz vs 6.078 ± 0.881 Hz; *U* = 1,427; *p* = 0.071; Mann–Whitney test), coefficient of variation % (naive females vs naive males; 90.08 ± 6.509% vs 119.0 ± 12.79%; *U* = 1,557; *p* = 0.264; Mann–Whitney test), and spikes in the burst percentage (naive females vs naive males; 9.175 ± 2.312% vs 7.127 ± 1.958%; *U* = 1,654; *p* = 0.534; Mann–Whitney test) between females and males. ***D***, The correlation plot reporting the burst rate against the spontaneous firing rate. Neurons without burst activity were excluded in this plot (naive females, *n* = 39 cells; naive males, *n* = 32 cells). Dark purple and dark green points showed the average value, respectively, as females and males.

We isolated the activity of 52 LHb neurons in females (from six mice) and 68 neurons in males (from six mice) that were located throughout the entire LHb territory (Fig. 1*A*,*B*). Consistent with previous reports, LHb neurons presented spontaneous action potentials in regular and irregular or with intermingled burst patterns (Fig. 1*B*; Kowski et al., 2009; Lecca et al., 2017; Yang et al., 2018; Cerniauskas et al., 2019; Congiu et al., 2019). The presence of those patterns was equally distributed in female and male mice (Fig. 1*B*).

We then analyzed the neuronal firing features, comparing values between females and males (Fig. 1*C*). As previously reported (Congiu et al., 2019), LHb neurons in isoflurane-anesthetized mice fired spontaneously at ∼6 Hz, with no significant difference between sexes (Fig. 1*C*). Although a recent report in rats indicated sex differences in the pattern of LHb neuronal activity (Bell et al., 2023), our analysis of the firing pattern, computed by the coefficient of variation of the interspike interval for each cell, did not reveal significant differences between females and males (Fig. 1*C*). LHb neurons are known to display burst firing, characterized by trains of action potentials in a rapid succession. This pattern may contribute to specific phenotypes, such as stress susceptibility versus resilience (Yang et al., 2018; Mondoloni et al., 2024). We compared the percentage of spikes in burst and the burst rate in our two experimental groups and found no significant differences (Fig. 1*C*,*D*).

Altogether, these data suggest that LHb spontaneous firing activity is comparable between sexes in virgin adult mice.

### LHb neuronal excitability in naive female and male mice

**Figure 2.**
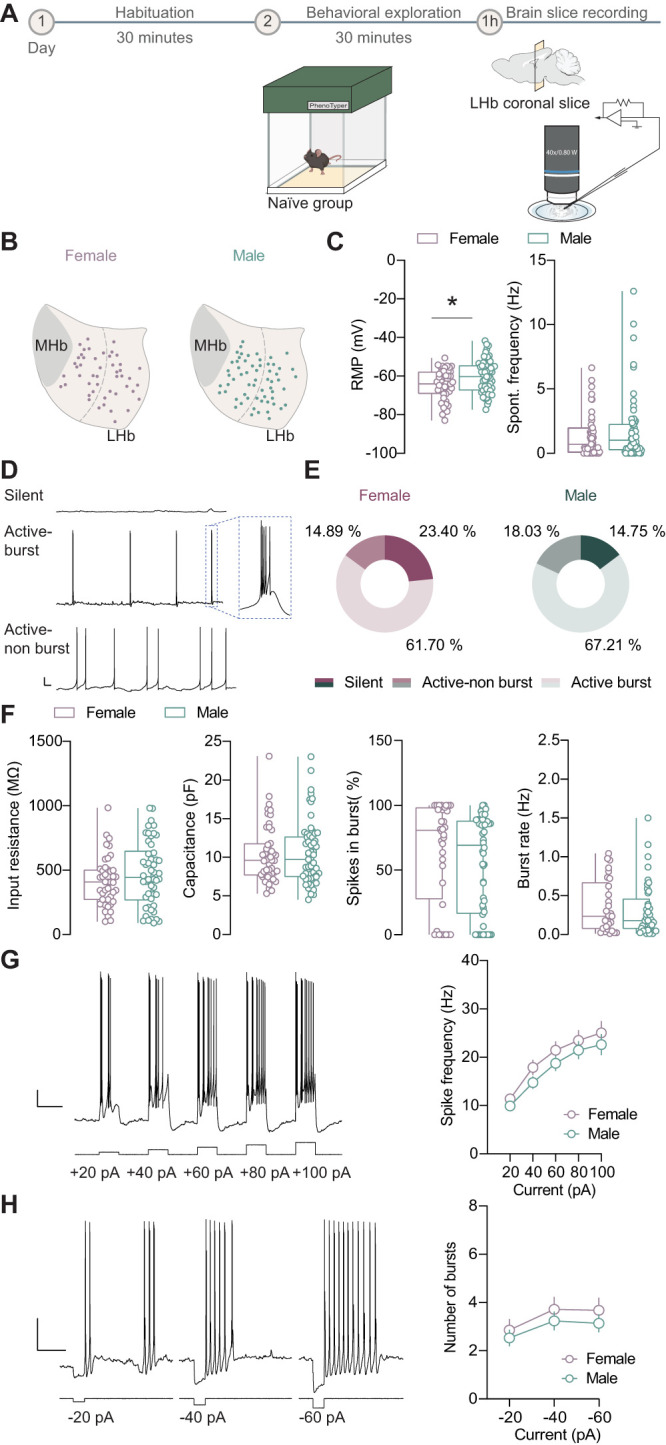
Ex vivo assessment of LHb neuronal activity in naive female and male mice. ***A***, Experimental timeline. Naive virgin females, *N* = 6 mice. Naive virgin males, *N* = 8 mice. ***B***, Territorial distribution of all recorded neurons from naive females (left) and males (right). The dashed line separates recorded neurons into medial and lateral territory. Naive virgin females, *n* = 47 cells. Naive virgin males, *n* = 61 cells (MHb, medial habenula). ***C***, Boxplots of RMP (left; naive females vs naive males; −64.24 ± 1.144 mV vs −60.15 ± 1.083 mV; *t* = 2.571; df = 106; *p = 0.012; unpaired *t* test) and spontaneous action potential frequency (right; naive females vs naive males; 1.375 ± 0.246 Hz vs 1.779 ± 0.323 Hz; *U* = 1,307; *p* = 0.432; Mann–Whitney test). ***D***, Example of different firing patterns from LHb neurons under RMP: silent, active neurons with burst firing or mixed with single spike in between and active neurons with single spike firing only (bar scale, 10 mV, 1 s). ***E***, The pie chart for distribution of firing patterns in naive female and male mice, numbers representing the percentage of each firing pattern (naive females vs naive males; *X*^2^ = 1.354; df = 2; *p* = 0.508; *χ*^2^ test). ***F***, Boxplots of input resistance (naive females vs naive males; 412.5 ± 29.60 MΩ vs 470.7 ± 35.73 MΩ; *t* = 1.226; df = 90; *p* = 0.224; unpaired *t* test). Naive virgin females, *n* = 42 cells. Naive virgin males, *n* = 50 cells. Capacitance (naive females vs naive males; 10.40 ± 0.545 pF vs 10.49 ± 0.523 pF; *U* = 1,429; *p* = 0.977; Mann–Whitney test). Naive virgin females, *n* = 47 cells. Naive virgin males, *n* = 61 cells. Boxplots of spikes in the burst percentage (naive females vs naive males; 64.47 ± 6.504% vs 55.21 ± 5.255%; *U* = 766.5; *p* = 0.150; Mann–Whitney test). Naive virgin females, *n* = 36 cells. Naive virgin males, *n* = 52 cells. Boxplot of the burst rate (naive females vs naive males; 0.357 ± 0.062 Hz vs 0.314 ± 0.053 Hz; *U* = 547; *p* = 0.575; Mann–Whitney test). Naive virgin females, *n* = 29 cells. Naive virgin males, *n* = 41 cells. ***G***, Sample trace of a recorded cell under RMP (left top; scale bar, 20 mV, 1 s) and experimental depolarization protocol (left bottom). Plots of action potential frequency in response to different injected currents (right; *p* = 0.930; *F*_(4,424)_ = 0.215; two-way ANOVA RM). Naive virgin females, *n* = 47 cells. Naive virgin males, *n* = 61 cells. ***H***, Sample trace of a recorded cell under RMP (left top) (scale bar, 20 mV, 2 s) and experimental hyperpolarization protocol (left bottom). The plot reporting the number of bursts after hyperpolarization in response to different injected currents (right; *p* = 0.811; *F*_(2,212)_ = 0.210; two-way ANOVA RM). Naive virgin females, *n* = 47 cells. Naive virgin males, *n* = 61 cells. See Extended Data Figure 2-1 and 2-2 for more details.

Although our in vivo assessment did not reveal differences in the spontaneous firing of LHb neurons, we next sought to compare the neuronal properties and excitability of LHb cells in naive female and male mice. In acute brain slices prepared 1 h after exposure to the behavioral arena (see Materials and Methods), we isolated LHb neurons in whole-cell current–clamp configuration (Fig. 2*A*). Recorded neurons were distributed throughout the LHb territory comparably between females and males (Fig. 2*B*).

10.1523/ENEURO.0414-24.2025.f2-1 Figure 2-1 **Lateral habenula territories and neuronal properties of naïve mice** Data were analyzed based on the separation indicated in figure 2B. Naïve virgin females: N= 6 mice. Naïve virgin males: N= 8 mice. (A-H): medial territory. (A) Boxplot of RMP (naïve females vs naïve males; -65.61 ± 1.724 mV vs -60.47 ± 1.342 mV; t = 2.385, df = 53, *p = 0.021, unpaired t-test). Naïve virgin females: n= 23 cells. Naïve virgin males: n= 32 cells. (B) Boxplot of spontaneous action potential frequency (naïve females vs naïve males; 1.407 ± 0.326 Hz vs 1.313 ± 0.276 Hz; U = 345.5, p = 0.706, Mann-Whitney test). Naïve virgin females: n= 23 cells. Naïve virgin males: n= 32 cells. (C) Boxplot of input resistance (naïve females vs naïve males; 440.8 ± 50.54 MΩ vs 479.5 ± 50.19 MΩ; t = 0.527, df = 44, p = 0.600, unpaired t-test). Naïve virgin females: n= 19 cells. Naïve virgin males: n= 27 cells. (D) Boxplot of capacitance (naïve females vs naïve males; 9.619 ± 0.698 pF vs 11.320 ± 0.749 pF; U = 281, p = 0.140, Mann-Whitney test). Naïve virgin females: n= 23 cells. Naïve virgin males: n= 32 cells. (E) Boxplot of spikes in burst % (naïve females vs naïve males; 63.91 ± 9.553 % vs 60.69 ± 6.972 %, U = 212, p = 0.427, Mann-Whitney test). Naïve virgin females: n= 19 cells. Naïve virgin males: n= 26 cells. (F) Boxplot of burst rate (naïve females vs naïve males; 0.417 ± 0.094 Hz vs 0.341 ± 0.070 Hz, U = 149.5, p = 0.641, Mann-Whitney test). Naïve virgin females: n= 15 cells. Naïve virgin males: n= 22 cells. (G) Plot of action potential frequency in response to different injected currents (p = 0.799, F(4,212) = 0.414, Two-way ANOVA RM). Naïve virgin females: n= 23 cells. Naïve virgin males: n= 32 cells. (H) Plot reporting number of bursts after hyperpolarization in response to different injected currents (p = 0.300, F(2,106) = 1.218, Two-way ANOVA RM). Naïve virgin females: n= 23 cells. Naïve virgin males: n= 32 cells. (I-P): lateral territory. (I) Boxplot of RMP (naïve females vs naïve males; -62.93 ± 1.500 mV vs -59.80 ± 1.756 mV; t = 1.326, df = 51, p = 0.191, unpaired t-test). Naïve virgin females: n= 24 cells. Naïve virgin males: n= 29 cells. (J) Boxplot of spontaneous action potential frequency (naïve females vs naïve males; 1.344 ± 0.374 Hz vs 2.292 ± 0.600 Hz; U = 259, p = 0.112, Mann-Whitney test). Naïve virgin females: n= 24 cells. Naïve virgin males: n= 29 cells. (K) Boxplot of input resistance (naïve females vs naïve males; 389.1 ± 34.65 MΩ vs 460.4 ± 51.74 MΩ; t = 1.145, df = 44, p = 0.258, unpaired t-test). Naïve virgin females: n= 23 cells. Naïve virgin males: n= 23 cells. (L) Boxplot of capacitance (naïve females vs naïve males; 11.160 ± 0.816 pF vs 9.568 ± 0.701 pF; U = 264.5, p = 0.138, Mann-Whitney test). Naïve virgin females: n= 24 cells. Naïve virgin males: n= 29 cells. (M) Boxplot of spikes in burst % (naïve females vs naïve males; 65.09 ± 9.010 % vs 49.72 ± 7.852 %, U = 173.5, p = 0.240, Mann-Whitney test). Naïve virgin females: n= 17 cells. Naïve virgin males: n= 26 cells. (N) Boxplot of burst rate (naïve females vs naïve males; 0.292 ± 0.080 Hz vs 0.282 ± 0.083 Hz, U = 124, p = 0.753, Mann-Whitney test). Naïve virgin females: n= 14 cells. Naïve virgin males: n= 19 cells. (O) Plot of action potential frequency in response to different injected currents (p = 0.860, F(4,204) = 0.327, Two-way ANOVA RM). Naïve virgin females: n= 24 cells. Naïve virgin males: n= 29 cells. (P) Plot reporting number of bursts after hyperpolarization in response to different injected currents (p = 0.808, F(2,102) = 0.214, Two-way ANOVA RM). Naïve virgin females: n= 24 cells. Naïve virgin males: n= 29 cells. Download Figure 2-1, TIF file.

10.1523/ENEURO.0414-24.2025.f2-2 Figure 2-2 ***Ex vivo* assessment of LHb neuronal activity in home-cage wild type female and male mice** (A) Experimental timeline. All mice were sacrificed, and brains were sliced into coronal sections directly after being taken out of their home-cage. WT females: N= 2 mice. WT males: N= 2 mice. (B) Boxplots of RMP (left) (WT females vs WT males; -64.17 ± 1.664 mV vs -61.77 ± 1.955 mV; t = 0.933, df = 38, p = 0.357, unpaired t-test) and spontaneous action potential frequency (right) (WT females vs WT males; 2.363 ± 0.560 Hz vs 1.139 ± 0.266 Hz; U = 144.5, p = 0.136, Mann-Whitney test). WT females: n= 20 cells. WT males: n= 20 cells. (C) Pie chart for distribution of firing patterns in naïve female and male mice, numbers representing the percentage of each firing pattern (WT females vs WT males; X2 = 1.146, df = 2, p = 0.930, Chi-Square test). WT females: n= 20 cells. WT males: n= 20 cells. (D) Boxplot of input resistance (WT females vs WT males; 434.3 ± 50.83 MΩ vs 446.3 ± 53.47 MΩ; U = 179, p = 0.989, Mann-Whitney test). WT females: n= 18 cells. WT males: n= 20 cells. Boxplot of capacitance (WT females vs WT males; 9.643 ± 0.734 pF vs 10.040 ± 0.840 pF; U = 192.5, p = 0.846, Mann-Whitney test). WT females: n= 20 cells. WT males: n= 20 cells. (E) Boxplot of spikes in burst % (WT females vs WT males; 46.40 ± 7.193 % vs 56.09 ± 8.525 %, U = 143, p = 0.277, Mann-Whitney test). WT females: n= 19 cells. WT males: n= 19 cells (left). (Right) burst rate (WT females vs WT males; 0.441 ± 0.130 Hz vs 0.308 ± 0.075 Hz, U = 94, p = 0.644, Mann-Whitney test), WT females: n= 15 cells. WT males: n= 14 cells. (F) Plot of action potential frequency in response to different injected currents (left). (p = 0.986, F(4,152) = 0.090, Two-way ANOVA RM). Plot reporting number of bursts after hyperpolarization in response to different injected currents (right) (p = 0.441, F(2,76) = 0.828, Two-way ANOVA RM). WT females: n= 20 cells. WT males: n= 20 cells. Download Figure 2-2, TIF file.

We found that the RMP differed between females and males, with females showing more hyperpolarized value (naive females vs naive males; −64.24 ± 1.144 mV vs −60.15 ± 1.083 mV; Fig. 2*C*). However, this difference did not affect the spontaneous firing frequency (Fig. 2*C*) and the firing pattern distribution between sexes (Fig. 2*D,E*). At their RMP, LHb cells in both sexes displayed a comparable distribution of silent cells, active cells with bursts, and active cells without bursts (Fig. 2*E*). We then further analyzed the input resistance and the cell capacitance, founding comparable averages between groups (Fig. 2*F*). Finally, we analyzed the percentage of spikes in burst, and the burst rate between sexes still obtained comparable values (Fig. 2*F*).

Next, we investigated LHb cell excitability in female and male mice by injecting incremental steps of depolarizing currents and simultaneously measuring evoked action potentials. LHb neurons fired more action potentials in response to incremental steps of injected currents, as previously demonstrated in male animals (Tchenio et al., 2017; Flerlage et al., 2024), and this response was not different when compared with LHb cells recorded in virgin females (Fig. 2*G*).

LHb neurons can exhibit bursts in response to hyperpolarizing currents (Wilcox et al., 1988; Yang et al., 2018; Mondoloni et al., 2024). We examined the number of bursts evoked by different steps of hyperpolarizing currents in LHb cells from both sexes. As shown in Figure 2*H*, LHb neurons displayed an increasing number of bursts in response to negative currents, with no significant difference between sexes.

Previous studies have shown that neurons can exhibit different properties based on their spatial organization within the LHb. For instance, sex-based genetic differences were recently highlighted in the medial territory of this region (Lecca et al., 2023). We then compared parameters based on the location of the recorded cells within the medial and lateral territories of the LHb (Extended Data Fig. 2-1). No differences in firing activity properties emerged between sexes for cells located in either the medial or lateral LHb territory (Extended Data Fig. 2-1). Finally, to understand whether the arena exposure (see Materials and Methods) led to potential adaptations, we performed experiments in a separate cohort of mice naive to any experience. No differences emerged in cell firing properties between females and males (Extended Data Fig. 2-2). In conclusion, these data, in line with the in vivo experiments, show comparable functional firing activity between virgin female and male mice in acute brain slices.

### Sex-divergent behaviors in virgin mice exposed to pups

Considering that the firing activity and neuronal excitability at the baseline in the LHb were not affected by sex, we investigated whether behavioral exposure to pups could induce specific sex-dependent adaptations in LHb function.

**Figure 3.**
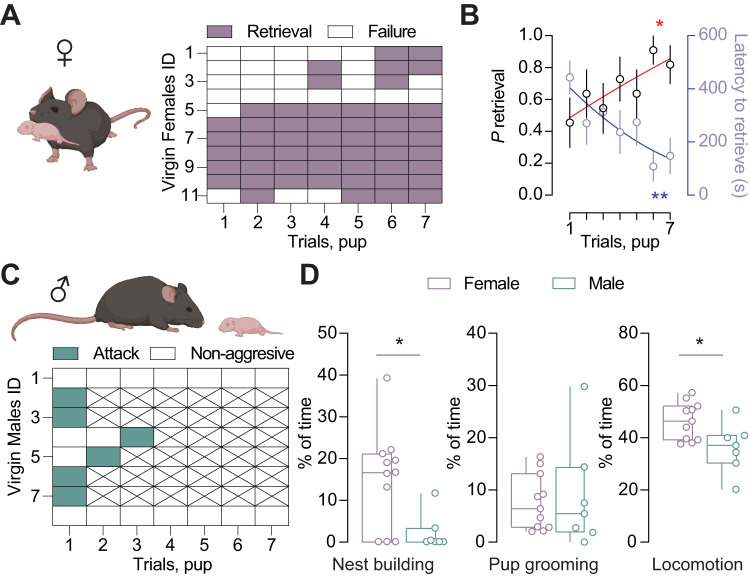
Behavioral sexual dimorphism of adult–newborn interaction in virgin mice. ***A***, Representative drawings depicting a virgin female during pup retrieval. Females started to take care of pups left outside the nest area and performed pup retrieval behaviors by picking pup back to nest. Binary map of virgin females showing the success (purple) and failure (white) to retrieve pups (left). *N* = 11 mice. ***B***, Retrieval probability (*n*_trials _= 7; *p = 0.049; *F*_(6,60) _= 2.911; one-way ANOVA RM) and the latency to retrieve (*n*_trials _= 7; **p = 0.002; *F*_(6,60) _= 6.006; one-way ANOVA RM with sigmoidal fit). ***C***, Illustration of a virgin male mouse interacting with a pup. Males were mostly aggressive or even attacked pups. Binary map of virgin males that attack the pup (teal) or nonaggressive (white), *N* = 8 mice. ***D***, Mice display a variety of parental actions such as nest building and pup grooming, Comparison between females and males in these activities during the experiment. One virgin male was removed due to the short latency to attack the pup right after the experiment began (the percentage of time spent in nest building, females vs males; 15.17 ± 3.557% vs 2.182 ± 1.642%; *U* = 15; *p = 0.029; Mann–Whitney test; the percentage of time spent in pup grooming, females vs males; 7.594 ± 1.596% vs 8.816 ± 3.925%; *t* = 0.332; df = 16; *p* = 0.744; unpaired *t* test; the percentage of total moving time during the experiment, females vs males; 46.40 ± 2.162% vs 36.20 ± 3.574%; *t* = 2.603; df = 16; *p = 0.019; unpaired *t* test; females, *N* = 11 mice; males, *N* = 7 mice).

First, we compared virgin females and males in their behavioral responses when exposed to pups (Fig. 3*A*,*C*). A series of seven pups (foreign to the adult, ranging from P2 to 6) were placed in a corner opposite to the nest for a total of 11 females (see Materials and Methods). Each pup was exposed for a maximum of 10 min before being removed, unless the female retrieved the pup to the nest. In line with previous reports, virgin females displayed pup retrieval behavior that improved with successive exposures. Specifically, they showed an increased probability and shorter latency to retrieve pups from the first to the seventh exposure, a phenomenon known as pup sensitization (Stolzenberg and Rissman, 2011), (Fig. 3*B*). When the same experiment was conducted using adult virgin males, we observed a remarkable difference in behavior: six out of eight males attacked the pup (Fig. 3*C*). Further analysis revealed that females spent significantly more time in nest building, while the time spent for pup grooming was comparable between sexes (Fig. 3*D*). Another notable difference was the total amount of time spent moving, with females being more active than males, potentially indicating that females had a greater interest in the pups (Fig. 3D). These results corroborate previous findings (Fang et al., 2018; Kohl et al., 2018) and underscored how sex differences reflect opposing adult–newborn interactions in virgin mice.

### LHb activity and excitability in virgin females and males after newborn interactions

**Figure 4.**
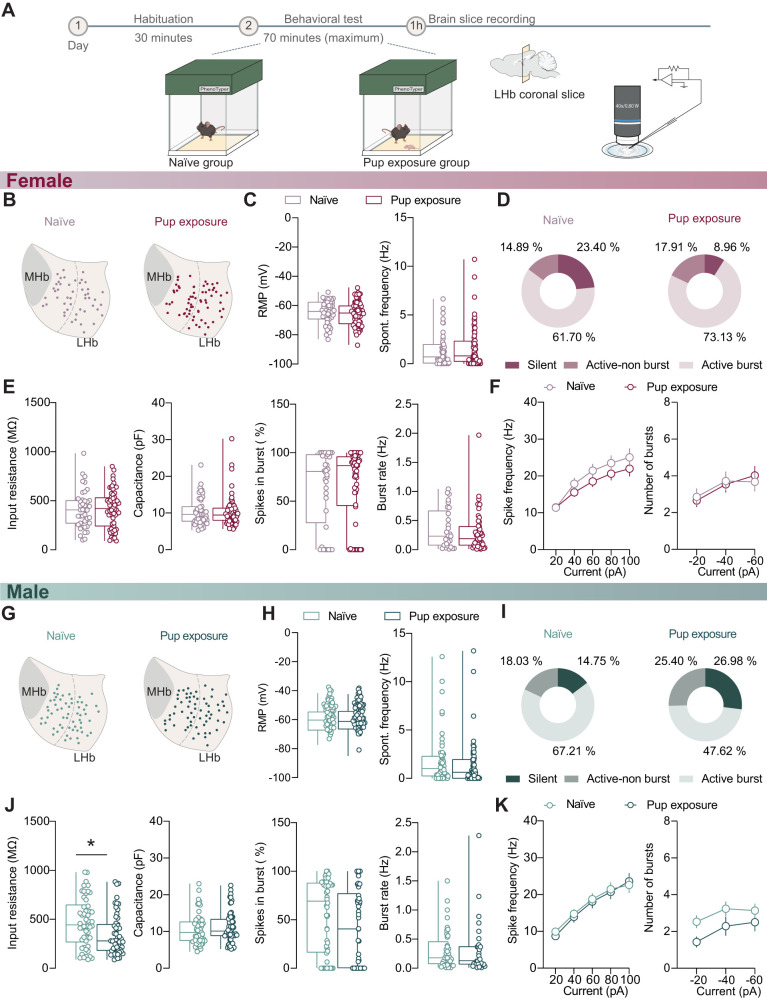
Comparison of LHb neuronal activity between sexes in naive and pup-exposed groups. ***A***, Experimental timeline of adult–newborn interaction and naive group. For female mice, naive group, *N* = 6 mice. Pup exposure group, *N* = 8 mice. ***B***, Territorial distribution of all recorded neurons from virgin females, naive group (left) and pup exposure group (right). the dashed line separates recorded neurons into medial and lateral territory. Naive group, *n* = 47 cells. Pup exposure group, *n* = 67 cells (MHb, medial habenula). ***C***, Boxplots of RMP (left; naive vs pup exposure; −64.24 ± 1.144 mV vs −65.63 ± 1.044 mV; *t* = 0.883; df = 112; *p* = 0.379; unpaired *t* test) and spontaneous action potential frequency (right; naive vs pup exposure; 1.375 ± 0.246 Hz vs 1.688 ± 0.261 Hz; *U* = 1,428; *p* = 0.401; Mann–Whitney test). Naive group, *n* = 47 cells. Pup exposure group, *n* = 67 cells. ***D***, Pie charts reporting the distribution of different firing patterns between naive and pup exposure group (*X*^2^ = 4.546; df = 2; *p* = 0.103; *χ*^2^ test). Naive group, *n* = 47 cells. Pup exposure group, *n* = 67 cells. ***E***, The boxplot of input resistance (naive vs pup exposure; 412.5 ± 29.60 MΩ vs 414.0 ± 23.93 MΩ; *t* = 0.041, df = 102, *p* = 0.968, unpaired *t* test). Naive group, *n* = 47 cells. Pup exposure group, *n* = 62 cells. Boxplot of capacitance (naive vs pup exposure; 10.40 ± 0.545 pF vs 10.31 ± 0.493 pF; *U* = 1,559; *p* = 0.930; Mann–Whitney test). Naive group, *n* = 47 cells. Pup exposure group, *n* = 67 cells. The boxplot of spikes in the burst percentage (naive vs pup exposure; 64.47 ± 6.504% vs 66.70 ± 4.881%; *U* = 1,089; *p* = 0.945; Mann–Whitney test). Naive group, *n* = 36 cells. Pup exposure group, *n* = 61 cells. Boxplot of the burst rate (naive vs pup exposure; 0.357 ± 0.062 Hz vs 0.307 ± 0.050 Hz; *U* = 656.6; *p* = 0.581; Mann–Whitney test). Naive group, *n* = 29 cells. Pup exposure group, *n* = 49 cells. ***F***, Plots of action potential frequency under depolarization protocol (left; *p* = 0.388; *F*_(4,488)_ = 1.037; two-way ANOVA RM) and the number of bursts induced by hyperpolarization protocol (right; *p* = 0.350; *F*_(2,224)_ = 1.054; two-way ANOVA RM) between groups from female mice. Naive group, *n* = 47 cells. Pup exposure group, *n* = 67 cells. For male mice, naive group, *N* = 8 mice. Pup exposure group, *N* = 8 mice. ***G***, Same as ***B*** but in males, naive group, *n* = 61 cells. Pup exposure group, *n* = 63 cells. ***H***, Boxplots of RMP (left; naive vs pup exposure; 60.15 ± 1.083 mV vs −60.51 ± 1.083 mV; *t* = 0.236; df = 122; *p* = 0.814; unpaired *t* test) and spontaneous action potential frequency (right; naive vs pup exposure; 1.779 ± 0.323 Hz vs 1.400 ± 0.296 Hz; *U* = 1,656; *p* = 0.183; Mann–Whitney test). ***I***, Same as ***D*** but in males (*X*^2^ = 5.061; df = 2; *p* = 0.080; *χ*^2^ test). ***J***, The boxplot of input resistance (naive vs pup exposure; 470.7 ± 35.73 MΩ vs 348.4 ± 28.45 MΩ; *U* = 966; *p = 0.012; Mann–Whitney test). Naive group, *n* = 50 cells. Pup exposure group, *n* = 54 cells. The boxplot of capacitance (naive vs pup exposure; 10.49 ± 0.523 pF vs 11.06 ± 0.531 pF; *U* = 1,750; *p* = 0.392; Mann–Whitney test). Naive group, *n* = 61 cells. Pup exposure group, *n* = 63 cells. The boxplot of spikes in the burst percentage (naive vs pup exposure; 55.21 ± 5.255% vs 41.77 ± 5.760%; *U* = 959.5; *p* = 0.089; Mann–Whitney test). Naive group, *n* = 52 cells. Pup exposure group, *n* = 46 cells. The boxplot of the burst rate (naive vs pup exposure; 0.314 ± 0.053 Hz vs 0.326 ± 0.086 Hz; *U* = 567.5; *p* = 0.584; Mann–Whitney test). Naive group, *n* = 41 cells. Pup exposure group, *n* = 30 cells. ***K***, Plots of action potential frequency under depolarization protocol (left; *p* = 0.765; *F*_(4,488)_ = 0.461; two-way ANOVA RM) and the number of bursts induced by hyperpolarization protocol (right; *p* = 0.381; *F*_(2,244)_ = 0.970; two-way ANOVA RM) between groups from male mice. Naive group, *n* = 61 cells. Pup exposure group, *n* = 63 cells. See Extended Data Figures 4-1, 4-2, 4-3, and 4-4 for more details.

LHb activity adapts after various behavioral experiences (Kang et al., 2017; Tchenio et al., 2017; Cerniauskas et al., 2019; Mondoloni et al., 2024). Could the adult–newborn encounter leave cellular traces on LHb function considering that LHb activity is recruited during pup interactions and retrievals in virgin females (Lecca et al., 2023)? We prepared acute LHb-containing brain slices 1 h after the mice experienced interactions with pups (Fig. 4*A*). Recordings from females that exhibited parental behaviors, including pup retrieval (Figs. 3*A*, 4*B*), were examined throughout the LHb and compared with naive controls (solely exposed to the behavioral arena; Figs. 2*B*–*H*, 4*A*,*B*). The RMP and spontaneous firing frequency were unaltered by pup exposure (Fig. 4*C*). *χ*^2^ test analysis of the firing pattern distribution revealed no statistical differences between the groups (Fig. 4*D*). Input resistance and cell capacitance as well as spontaneous burst properties were not altered by pup exposure (Fig. 4*E*). Furthermore, neither neuronal excitability in response to depolarizing currents nor the number of bursts in response to hyperpolarizing currents was affected by pup exposure (Fig. 4*F*). We next examined whether differences in LHb neuronal properties were emerging in specific LHb territories. No significant differences occurred across properties for cells in the medial or lateral aspects of LHb (Extended Data Fig. 4-1). Additionally, we assessed whether behavioral performance in the pup-exposed group and cell properties were related to one another. The plotting time spent nest building and pup grooming against RMP, input resistance, spontaneous firing rate, and spikes in burst and burst rate revealed no significant correlations across all mice employed nor when selecting top-performing retrievers (see Materials and Methods; Extended Data Fig. 4-2). Together, these findings suggest that 1 h after pup exposure, LHb excitability in virgin females remains unaltered, regardless of LHb territory or behavioral performance.

10.1523/ENEURO.0414-24.2025.f4-1 Figure 4-1 **Lateral habenula territories and neuronal properties of naïve and pup-exposed female mice** Data were analyzed based on the separation indicated in figure 4B. Naïve group: N= 6 mice. Pup exposure group: N= 8 mice. (A-H): medial territory. (A) Boxplot of RMP (naïve vs pup exposure; -65.61 ± 1.724 mV vs -67.07 ± 1.448 mV, t = 0.652, df = 53, p = 0.517, unpaired t-test). Naïve group: n= 23 cells. Pup exposure group: n= 32 cells. (B) Boxplot of spontaneous action potential frequency (naïve vs pup exposure; 1.407 ± 0.326 Hz vs 1.732 ± 0.426 Hz, U = 360.5, p = 0.902, Mann-Whitney test). Naïve group: n= 23 cells. Pup exposure group: n= 32 cells. (C) Boxplot of input resistance (naïve vs pup exposure; 440.8 ± 50.54 MΩ vs 402.2 ± 38.65 MΩ; t = 0.610, df = 48, p = 0.545, unpaired t-test). Naïve group: n= 19 cells. Pup exposure group: n= 31 cells. (D) Boxplot of capacitance (naïve vs pup exposure; 9.619 ± 0.698 pF vs 10.76 ± 0.787 pF; U = 309, p = 0.319, Mann-Whitney test). Naïve group: n= 23 cells. Pup exposure group: n= 32 cells. (E) Boxplot of spikes in burst % (naïve vs pup exposure; 63.91 ± 9.553 % vs 72.64 ± 7.249 %, U = 238, p = 0.548, Mann-Whitney test). Naïve group: n= 19 cells. Pup exposure group: n= 28 cells. (F) Boxplot of burst rate (naïve vs pup exposure; 0.417 ± 0.094 Hz vs 0.369 ± 0.092 Hz, U = 155.5, p = 0.621, Mann-Whitney test). Naïve group: n= 15 cells. Pup exposure group: n= 23 cells. (G) Plot of action potential frequency in response to different injected currents (p = 0.200, F(4,212) = 1.510, Two-way ANOVA RM). Naïve group: n= 23 cells. Pup exposure group: n= 32 cells. (H) Plot reporting number of bursts after hyperpolarization in response to different injected currents (p = 0.765, F(2,106) = 0.268, Two-way ANOVA RM). Naïve group: n= 23 cells. Pup exposure group: n= 32 cells. (I-P): lateral territory. (I) Boxplot of RMP (naïve vs pup exposure; -62.93 ± 1.500 mV vs -64.31 ± 1.480 mV; t = 0.632, df = 57, p = 0.530, unpaired t-test). Naïve group: n= 24 cells. Pup exposure group: n= 35 cells. (J) Boxplot of spontaneous action potential frequency (naïve vs pup exposure; 1.344 ± 0.374 Hz vs 1.648 ± 0.318 Hz; U = 340.5, p = 0.222, Mann-Whitney test). Naïve group: n= 24 cells. Pup exposure group: n= 35 cells. (K) Boxplot of input resistance (naïve vs pup exposure; 389.1 ± 34.65 MΩ vs 425.8 ± 28.73 MΩ; t = 0.821, df = 52, p = 0.415, unpaired t-test). Naïve group: n= 23 cells. Pup exposure group: n= 31 cells. (L) Boxplot of capacitance (naïve vs pup exposure; 11.160 ± 0.816 pF vs 9.891 ± 0.615 pF; U = 334, p = 0.187, Mann-Whitney test). Naïve group: n= 24 cells. Pup exposure group: n= 35 cells. (M) Boxplot of spikes in burst % (naïve vs pup exposure; 65.09 ± 9.010 % vs 61.66 ± 6.574 %, U = 259, p = 0.665, Mann-Whitney test). Naïve group: n= 17 cells. Pup exposure group: n= 33 cells. (N) Boxplot of burst rate (naïve vs pup exposure; 0.292 ± 0.080 Hz vs 0.252 ± 0.045 Hz, U = 180.5, p = 0.972, Mann-Whitney test). Naïve group: n= 14 cells. Pup exposure group: n= 26 cells. (O) Plots of action potential frequency in response to different injected currents (p = 0.648, F(4,228) = 0.622, Two-way ANOVA RM). Naïve group: n= 24 cells. Pup exposure group: n= 35 cells. (P) Plot reporting number of bursts after hyperpolarization in response to different injected currents (p = 0.363, F(2,114) = 1.024, Two-way ANOVA RM). Naïve group: n= 24 cells. Pup exposure group: n= 35 cells. Download Figure 4-1, TIF file.

10.1523/ENEURO.0414-24.2025.f4-2 Figure 4-2 **Correlation between behaviors and electrophysiological data in female mice** (A) A correlation matrix illustrating the relationship between behaviors—pup grooming and nest building—and electrophysiological parameters: resting membrane potential (RMP), input resistance, spontaneous firing rate, spikes in bursts, and burst rate. The analysis involves the average electrophysiological data from each mouse and its behavioral time allocation, aggregated across a total of 8 females exposed to pups. Pearson correlation r value displayed. (B) *Ex vivo* data comparison between naïve females and females exhibiting high retrieval performance, naïve females: N= 6 mice, n= 47 cells, high retrieval: N=4 mice, n= 33 cells. Boxplot of spontaneous action potential frequency (naïve vs high retrieval; 1.375 ± 0.246 Hz vs 1.721 ± 0.421 Hz; U = 703, p = 0.481, Mann-Whitney test). Plot of action potential frequency in response to different injected currents (p = 0.077, F(4,312) = 2.132, Two-way ANOVA RM). Plot reporting number of bursts after hyperpolarization in response to different injected currents (p = 0.489, F(2,156) = 0.719, Two-way ANOVA RM). Download Figure 4-2, TIF file.

10.1523/ENEURO.0414-24.2025.f4-3 Figure 4-3 **Lateral habenula territories and neuronal properties of naïve and pup exposure male mice** Data were analyzed based on the separation indicated in figure 4G. Naïve group: N= 8 mice. Pup exposure group: N= 8 mice. (A-H): medial territory. (A) Boxplot of RMP (naïve vs pup exposure; -60.47 ± 1.342 mV vs -60.48 ± 1.389 mV, t = 0.005, df = 63, p = 0.996, unpaired t-test). Naïve group: n= 32 cells. Pup exposure group: n= 33 cells. (B) Boxplot of spontaneous action potential frequency (naïve vs pup exposure; 1.313 ± 0.276 Hz vs 1.350 ± 0.432 Hz, U = 488.5, p = 0.608, Mann-Whitney test). Naïve group: n= 32 cells. Pup exposure group: n= 33 cells. (C) Boxplot of input resistance (naïve vs pup exposure; 479.5 ± 50.19 MΩ vs 352.5 ± 39.17 MΩ; U = 298, p = 0.061, Mann-Whitney test). Naïve group: n= 27 cells. Pup exposure group: n= 31 cells. (D) Boxplot of capacitance (naïve vs pup exposure; 11.32 ± 0.749 pF vs 11.29 ± 0.695 pF; U = 526, p = 0.982, Mann-Whitney test). Naïve group: n= 32 cells. Pup exposure group: n= 33 cells. (E) Boxplot of spikes in burst % (naïve vs pup exposure; 60.69 ± 6.972 % vs 42.72 ± 7.447 %, U = 256, p = 0.090, Mann-Whitney test). Naïve group: n= 26 cells. Pup exposure group: n= 27 cells. (F) Boxplot of burst rate (naïve vs pup exposure; 0.341 ± 0.070 Hz vs 0.287 ± 0.122 Hz, U = 152, p = 0.216, Mann-Whitney test). Naïve group: n= 22 cells. Pup exposure group: n= 18 cells. (G) Plot of action potential frequency in response to different injected currents (p = 0.935, F(4,252) = 0.206, Two-way ANOVA RM). Naïve group: n= 32 cells. Pup exposure group: n= 33 cells. (H) Plot reporting number of bursts after hyperpolarization in response to different injected currents (p = 0.120, F(2,126) = 2.157, Two-way ANOVA RM). Naïve group: n= 32 cells. Pup exposure group: n= 33 cells. (I-P): lateral territory. (I) Boxplot of RMP (naïve vs pup exposure; -59.80 ± 1.756 mV vs -60.55 ± 1.711 mV; t = 0.306, df = 57, p = 0.761, unpaired t-test). Naïve group: n= 29 cells. Pup exposure group: n= 30 cells. (J) Boxplot of spontaneous action potential frequency (naïve vs pup exposure; 2.292 ± 0.600 Hz vs 1.454 ± 0.407 Hz; U = 347.5, p = 0.184, Mann-Whitney test). Naïve group: n= 29 cells. Pup exposure group: n= 30 cells. (K) Boxplot of input resistance (naïve vs pup exposure; 460.4 ± 51.74 MΩ vs 342.9 ± 41.91 MΩ; U = 186, p = 0.087, Mann-Whitney test). Naïve group: n= 23 cells. Pup exposure group: n= 23 cells. (L) Boxplot of capacitance (naïve vs pup exposure; 9.568 ± 0.701 pF vs 10.80 ± 0.821 pF; U = 359.5, p = 0.256, Mann-Whitney test). Naïve group: n= 29 cells. Pup exposure group: n= 30 cells. (M) Boxplot of spikes in burst % (naïve vs pup exposure; 49.72 ± 7.852 % vs 40.42 ± 9.316 %, U = 208.5, p = 0.376, Mann-Whitney test). Naïve group: n= 26 cells. Pup exposure group: n= 19 cells. (N) Boxplot of burst rate (naïve vs pup exposure; 0.282 ± 0.083 Hz vs 0.384 ± 0.117 Hz, U = 99.5, p = 0.568, Mann-Whitney test). Naïve group: n= 19 cells. Pup exposure group: n= 12 cells. (O) Plots of action potential frequency in response to different injected currents (p = 0.876, F(4,228) = 0.303, Two-way ANOVA RM). Naïve group: n= 29 cells. Pup exposure group: n= 30 cells. (P) Plot reporting number of bursts after hyperpolarization in response to different injected currents (p = 0.758, F(2,114) = 0.278, Two-way ANOVA RM). Naïve group: n= 29 cells. Pup exposure group: n= 30 cells. Download Figure 4-3, TIF file.

10.1523/ENEURO.0414-24.2025.f4-4 Figure 4-4 **Correlation between behaviors and electrophysiological data in male mice** (A) A correlation matrix illustrating the relationship between behaviors—pup grooming and nest building—and electrophysiological parameters: resting membrane potential (RMP), input resistance, spontaneous firing rate, spikes in bursts, and burst rate. The analysis involves the average electrophysiological data from each mouse and its behavioral time allocation, aggregated across a total of 8 males exposed to pups. One male was excluded due to immediately aggression toward pups right after experiment started. Pearson correlation r value displayed. (grooming the pup vs spontaneous firing rate: *p = 0.012) (B) *Ex vivo* data comparison between naïve males and males exhibiting attack behavior toward newborn. Naïve males: N= 8 mice, n= 61 cells, attacker: N=6 mice, n= 47 cells. Boxplot of spontaneous action potential frequency (naïve vs attacker; 1.779 ± 0.323 Hz vs 1.462 ± 0.372 Hz; U = 1216, p = 0.177, Mann-Whitney test). Plots of action potential frequency in response to different injected currents (p = 0.393, F(4,424) = 1.027, Two-way ANOVA RM). Plot reporting number of bursts after hyperpolarization in response to different injected currents (p = 0.354, F(2,212) = 1.044, Two-way ANOVA RM). Download Figure 4-4, TIF file.

In a separate set of experiments, we tested male mice exposed to pups and compared their neuronal properties with a control group solely exposed to the behavioral arena (Figs. 2*B*–*H*, 4*A*). The territorial distribution of the recorded cells in the LHb was similar between the two experimental groups (Fig. 4*G*). Using a current-clamp configuration, we measured the RMP and the spontaneous frequency of action potentials in LHb cells finding no significant differences between groups (Fig. 4*H*). Similarly to females, LHb cells in males exposed to pups did not show any differences in firing pattern distribution (Fig. 4*I*). In males, pup exposure led to a reduction in input resistance leaving instead unaltered cell capacitance, spontaneous burst, excitability, and hyperpolarization-induced rebound burst (Fig. 4*J*,*K*). No adaptations in firing properties were observed when the analysis was confined to specific LHb territories (Extended Data Fig. 4-3). Notably, a positive correlation emerged between time spent pup grooming and spontaneous frequency (Extended Data Fig. 4-4). However, comparing naive versus males showing pup-attack indicated no differences between groups (Extended Data Fig. 4-4).

In summary, these data suggest that despite experiencing pups trigger profound sex-based divergent behavior, this does not lead to firing adaptations in LHb neuronal output.

## Discussion

In this study, we aimed to investigate whether sex differentially controlled spontaneous activity, action potential patterns, and excitability of LHb neurons in virgin female and male mice, both under baseline conditions and after exposure to pups.

Our results revealed no significant changes in spontaneous firing rates, firing patterns, or neuronal excitability between sexes. The only difference observed was a more hyperpolarized RMP in females, which however did not translate into any further changes in neuronal activity or excitability. These changes in RMP may nevertheless be relevant for other aspects of LHb cell function as previously described in both physiological and pathological conditions (Cui et al., 2018; Takahashi et al., 2022).

These findings are interesting considering the existing anatomical and physiological evidence that suggest for sexual dimorphism in the LHb (Lonstein et al., 2005; Rigney et al., 2020; Liu et al., 2022). Indeed, a recent in vivo study in urethane-anesthetized rats revealed sex differences in LHb firing patterns with more regular firing in female (Bell et al., 2023). This discrepancy may emerge due to the different anesthetic employed or to species-specific differences between mice and rats. Transcriptomic data indicate sex differences in subpopulations of LHb cells (Lecca et al., 2023). This raises the scenario whereby sex-dependent functional differences may be localized to specific neuronal subsets and therefore nondetectable when looking at the global population. However, in our ex vivo recordings, the analysis based on cell location did not unravel any difference between sexes, limiting but not abolishing this possibility. A limitation of the present study is represented by the lack of control for the estrous cycle in female individuals. Estradiol fluctuations acting on estrogen receptors were reported to influence neuronal activity in diverse brain regions, directly or indirectly tuning LHb firing output (Li et al., 2015; Calvigioni et al., 2023). Future studies should examine the impact of the different phases of the estrus cycle on the firing properties of LHb cells in females. Despite previous reports suggesting sex-based differences in the LHb (Lonstein et al., 2005; Rigney et al., 2020; Bell et al., 2023; Li et al., 2023), our findings show no significant functional differences in basal firing activity between sexes. Nonetheless, we cannot exclude the possibility that sex differences at other levels may emerge for instance on excitatory/inhibitory balance, synaptic plasticity, or firing responses to external stimuli.

The prediction that adaptations in LHb firing activity may occur after adult–newborn interactions is in line with the established understanding that the LHb is critical for adult–newborn behaviors in virgin females (Felton et al., 1998; Lecca et al., 2023). LHb activity is essential for pup retrieval, and these neurons uniquely bridge aversion with parental actions, providing a neuronal substrate for negative reinforcement and parental behavior (Carta and Autry, 2023). Distress pup calls serve as aversive signals to virgin females who are willing to press a lever to turn off distress pup calls (Schiavo et al., 2020). LHb neurons respond with excitation to pup distress calls, guiding retrieval actions (Lecca et al., 2023).

However, our findings suggest that LHb activity during these behaviors is recruited on demand participating to the parental circuitry yet not representing a site of for long-term plasticity. Nevertheless, we cannot rule out potential firing adaptations that might occur at a different timescale than the one investigated in this study. Similarly, we cannot exclude that adaptations may occur at the level of synaptic strength and potentially at specific inputs. Future studies should address these questions.

In both virgin females and males, pup exposure left unaltered several neuronal properties, except for a reduction in input resistance observed after pup exposure in males. Importantly, neuronal input resistance relies, at least partly, on potassium channels. Experience-dependent plasticity of potassium conductance, including GIRKs, has been described in the LHb (Lecca et al., 2016). Thus, it is plausible that pup exposure might act on complementary signaling pathways specifically changing neuronal biophysical features.

For virgin males, pup-directed aggressive behavior is reported to involve multiple sensory stimuli, including salivary residues from the mother, the physical shape of pups, and an intact vomeronasal organ (VNO; Wu et al., 2014; Isogai et al., 2018) . Especially, after pup exposure, virgin males have higher c-Fos expression in the VNO compared with fathers, which are fully parental (Tachikawa et al., 2013). However, whether the LHb is active during virgin male-pup attacks remains to be explored. This is an interesting avenue of research as the LHb participates to other aspects of aggression, for instance, between adult males (Golden et al., 2016; Flanigan et al., 2020).

Pup-directed interactions require intact BNST-to-LHb circuit in virgin females (Lecca et al., 2023). While one alternative would be to explore BNST-specific input efficacy onto LHb in a sex-dependent fashion, it is worth noting that rabies-based monosynaptic mapping studies in female and male mice showed no differences in the number of BNST neurons projecting their axons to the LHb (Liu et al., 2022; Huang et al., 2024).

It is therefore possible that other brain regions which are highly sexually dimorphic may play more significant roles in mediating the observed opposing adult–newborn interactions between females and males (Bayless and Shah, 2016; Zilkha et al., 2017). Indeed, while the LHb is crucial for specific aspects, the hypothalamic medial preoptic area and ventral hypothalamus, as well as the amygdala and extended amygdala, can be sites where baseline and plasticity differences may emerge in a sexually dimorphic fashion (Scott et al., 2015; Yang and Shah, 2016; Kohl et al., 2018; Chen et al., 2019).

In conclusion, this study offers relevant parameters related to the function of the LHb in female and male virgin mice and may steer future studies to better refine our knowledge on the circuit mechanisms underlying adult–newborn behaviors.

## Synthesis

Reviewing Editor: Mark Sheffield, University of Chicago Division of the Biological Sciences Decisions are customarily a result of the Reviewing Editor and the peer reviewers coming together and discussing their recommendations until a consensus is reached. When revisions are invited, a fact-based synthesis statement explaining their decision and outlining what is needed to prepare a revision will be listed below. The following reviewer(s) agreed to reveal their identity: ChiHye Chung.

The manuscript provides an in-depth examination of neuronal properties in the lateral habenula (LHb) of adult male and female mice, focusing on potential sex-specific and experience-dependent behavioral differences. Both reviewers acknowledge the significant effort and comprehensive nature of the electrophysiological recordings, recognizing the potential value of this study to the field. However, there are key areas that require further attention to ensure that the findings align with the conclusions drawn.

Reviewer 1 appreciates the rigorous approach and believes that the manuscript holds promise as an important contribution after revisions. Reviewer 2, while acknowledging the thorough experimental approach, raises concerns about the sufficiency of the current data to support the claim that LHb activity does not reflect sex-specific adult-newborn interactions. Both reviewers agree that additional clarification of the hypotheses, refinement of the claims, and potential adjustments to the title are necessary to align the manuscript with the data presented.

The reviewers suggest that the authors consider revising their claims to better reflect the current findings or include additional experimental evidence that demonstrates LHb activity during direct behavioral interactions. Incorporating more detailed analyses and methodological clarifications, along with discussions on hormonal influences and behavioral correlations, will strengthen the manuscript's contributions and impact.

In summary, a "Revise and Re-Review" decision is recommended if the authors choose to revise their claims, address the reviewers' methodological suggestions, and align their conclusions with the existing data. The detailed reviewer comments that follow will provide further guidance for these revisions.

Reviewer 1:

1) In vivo recordings

The in vivo recordings provide important insights into baseline LHb neural activity. These recordings constitute a rich dataset which can be explored a bit further. Below are my specific suggestions about additional analysis / visualization of the data:

a) I am curious about the medial-lateral distribution of the ephys parameters, especially with regard to findings from Lecca et al., 2023. It would be interesting to see where in terms of coordinates reside neurons exhibiting specific spiking patterns. For example, the authors could plot in different colors/symbols the individual neurons in Fig 1. A, right panel, depending on spiking pattern.

b) In order to make parallels between in vivo and slice recordings, I'd suggest to add pie charts of different spiking patterns in females vs males in Fig. 1B similar to Fig. 2E. I do not expect the distribution of irregular/regular/bursty cells to be necessarily similar between in vivo and ex vivo conditions.

c) It would be interesting to plot firing rate against burst frequency similar to Mondoloni et al., 2024 Fig. S5J.

2) Ex vivo recordings

The slice recordings can be described a bit more in detail. Suggestions below:

a) Plot additional parameters such as Ri, capacitance.

b) Plot burst rate and spikes in burst in order to make a parallel with the in vivo recordings in Fig.1.

c) Splitting the data into cells recorded within the medial and lateral divisions of the LHb might help unravel some potential differences.

3) Behavior

a) It would be intriguing to correlate behavior output (not only the exposure to pups itself) with cell properties. For example - compare cell properties in females which had high retrieval score / lower latency to retrieve vs cell properties in males which had lower latency to attack. Some potential differences might be uncovered when comparing individuals exhibiting drastically different behaviors, and excluding from the analysis animals which ignored pups. In contracts, maybe no difference in cell properties will be found in individuals of either sex which ignored pups?

b) In similar line of reasoning - do burst properties correlate to behavior?

Reviewer 2:

Major Comments:

1. The authors did not present a clear hypothesis on how LHb might be involved in sex-specific adult-newborn social interactions. Based on prior research, including expectations about LHb activation could enhance the readers' understanding.

2. The authors mentioned in the introduction that LHb exhibits significant sex differences in estrogen receptor expression. They also noted the potential involvement of hormones like estrogen and vasopressin in sex-dependent social behaviors. Although the authors anticipated that estrogen might affect sex-specific adult-newborn interactions, they did not control for this variable, arguing in the discussion that it could introduce excessive variance. However, experimental verification-such as measuring LHb neuronal responses to estrogen or reducing LHb-specific estrogen receptor expression-would strengthen the claim that LHb functions independently of hormone levels in regulating these interactions.

Additionally, since exposure to the pups lasted (at most) 70 minutes, the estrogen cycle could play a significant role. Notably, many of the referenced studies investigate the role of LHb concerning hormonal changes, yet there seems to be no consideration of estrogen in this study (Corodimas et al., 1992; Matthews-Felton et al., 1995).

3. Overall, the data presented does not sufficiently support the title "Neuronal activity in the Lateral Habenula does not reflect sex-specific adult-newborn interactions in virgin mice." The study only shows resting membrane potential (RMP), firing patterns, and changes due to current injection, but the proportion of LHb burst-firing cells in the naïve state appears to be higher than previously reported (Fig. 2E) (Yan Yang et al., 2018). It raises the question of whether the one hour spent in the novel open arena might have increased anxiety levels, potentially resulting in more basal burst-firing cells and obscuring subsequent changes. For the reasons above, I believe the LHb activity in the basal state may not have been accurately measured.

4. The study primarily relied on spontaneous neuronal activity as a functional indicator. Previous studies suggest that specific neural pathways, such as BNST-LHb or MPOA-LHb, involve social behaviors like parental care. Since these pathways may play a more direct role than spontaneous activity, a synaptic-level analysis is needed. The integrated signals measured in the study might not reveal meaningful behavioral associations, which could explain the lack of correlation between LHb activity and behavior.

5. Observing the behavior of virgin males, they tend to attack in the early trials, leading to less exposure to the pups. This raises the question of whether the males perceived this protocol as a newborn interaction. Is it possible that the study measured the LHb's response to aggression rather than true interaction?

6. The authors measured neuronal activity after the behavioral task, not during the animals' engagement in social behavior. Measuring at such a time point may only capture limited changes. What they assessed reflects LHb function altered by the pup retrieval task but does not reveal LHb's real-time involvement during sex-specific adult-newborn interactions. To argue convincingly that LHb is not involved in these interactions, they should confirm the absence of sex-based differences in LHb function during the actual behavior. Additionally, experimental manipulations would be necessary to verify the causal relationship between LHb activity and behavior.

Minor Comments:

Figure 1C: The distribution of data points between males and females appears different. Conducting a Kolmogorov-Smirnov test could reveal significant distributional differences that might not be apparent through mean comparisons alone.

Figure 4: Gender is only indicated by color, which may reduce clarity. Adding text labels alongside the colors would improve readability.
