# Supplementary figures and images for: Neuronal Properties in the Lateral Habenula and Adult–Newborn Interactions in Virgin Female and Male Mice
Source: eNeuro. 2025 Feb 11;12(2):ENEURO.0414-24.2025. doi: 10.1523/ENEURO.0414-24.2025 (PMC11839275; doi:10.1523/ENEURO.0414-24.2025)

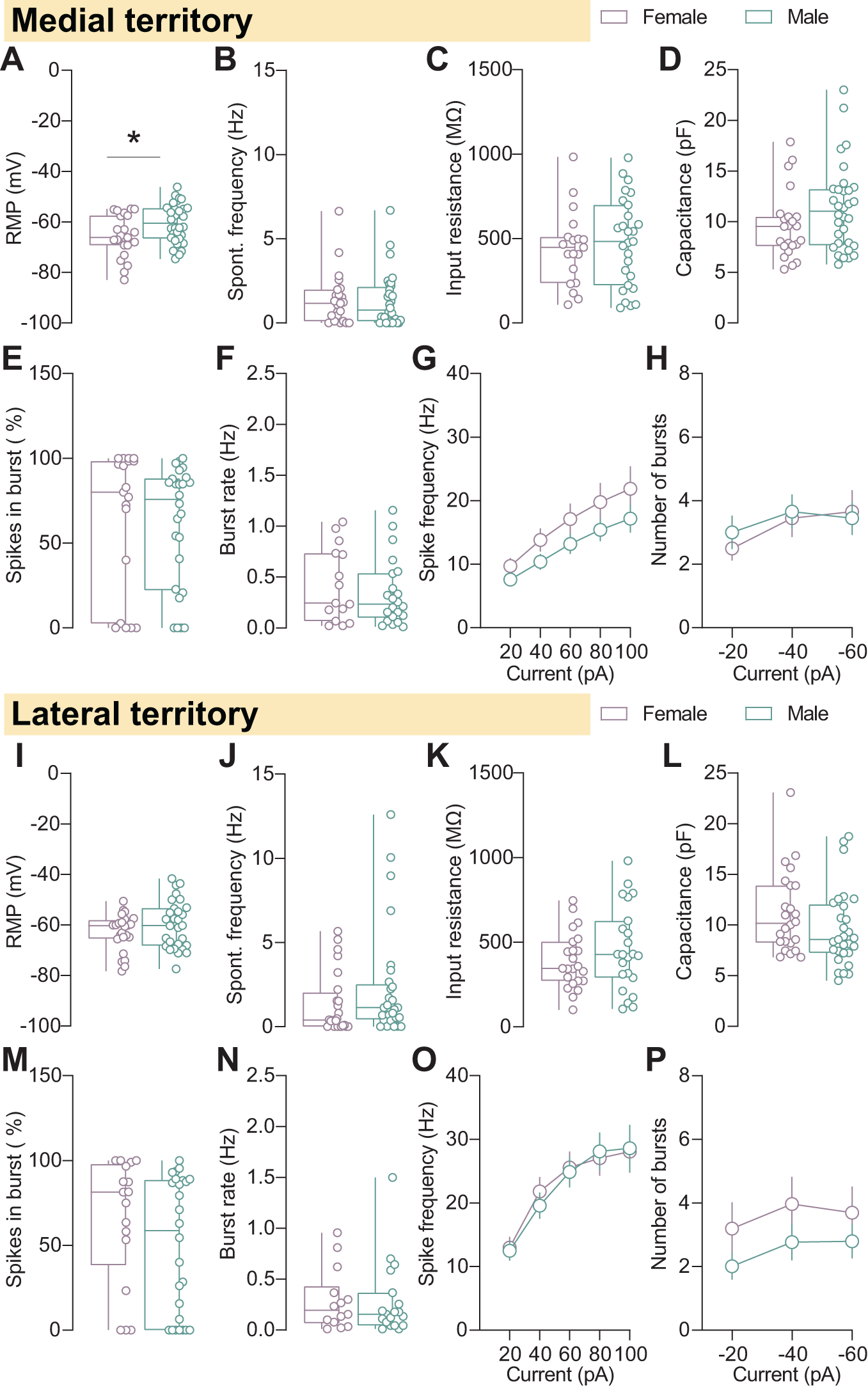

Supplement: Figure 2-1 — Lateral habenula territories and neuronal properties of naïve mice Data were analyzed based on the separation indicated in figure 2B. Naïve virgin females: N= 6 mice. Naïve virgin males: N= 8 mice. (A-H): medial territory. (A) Boxplot of RMP (naïve females vs naïve males; -65.61 ± 1.724 mV vs -60.47 ± 1.342 mV; t = 2.385, df = 53, *p = 0.021, unpaired t-test). Naïve virgin females: n= 23 cells. Naïve virgin males: n= 32 cells. (B) Boxplot of spontaneous action potential frequency (naïve females vs naïve males; 1.407 ± 0.326 Hz vs 1.313 ± 0.276 Hz; U = 345.5, p = 0.706, Mann-Whitney test). Naïve virgin females: n= 23 cells. Naïve virgin males: n= 32 cells. (C) Boxplot of input resistance (naïve females vs naïve males; 440.8 ± 50.54 MΩ vs 479.5 ± 50.19 MΩ; t = 0.527, df = 44, p = 0.600, unpaired t-test). Naïve virgin females: n= 19 cells. Naïve virgin males: n= 27 cells. (D) Boxplot of capacitance (naïve females vs naïve males; 9.619 ± 0.698 pF vs 11.320 ± 0.749 pF; U = 281, p = 0.140, Mann-Whitney test). Naïve virgin females: n= 23 cells. Naïve virgin males: n= 32 cells. (E) Boxplot of spikes in burst % (naïve females vs naïve males; 63.91 ± 9.553 % vs 60.69 ± 6.972 %, U = 212, p = 0.427, Mann-Whitney test). Naïve virgin females: n= 19 cells. Naïve virgin males: n= 26 cells. (F) Boxplot of burst rate (naïve females vs naïve males; 0.417 ± 0.094 Hz vs 0.341 ± 0.070 Hz, U = 149.5, p = 0.641, Mann-Whitney test). Naïve virgin females: n= 15 cells. Naïve virgin males: n= 22 cells. (G) Plot of action potential frequency in response to different injected currents (p = 0.799, F(4,212) = 0.414, Two-way ANOVA RM). Naïve virgin females: n= 23 cells. Naïve virgin males: n= 32 cells. (H) Plot reporting number of bursts after hyperpolarization in response to different injected currents (p = 0.300, F(2,106) = 1.218, Two-way ANOVA RM). Naïve virgin females: n= 23 cells. Naïve virgin males: n= 32 cells. (I-P): lateral territory. (I) Boxplot of RMP (naïve females vs naïve males; -62.9 [file eneuro-12-ENEURO.0414-24.2025-s002.tif]

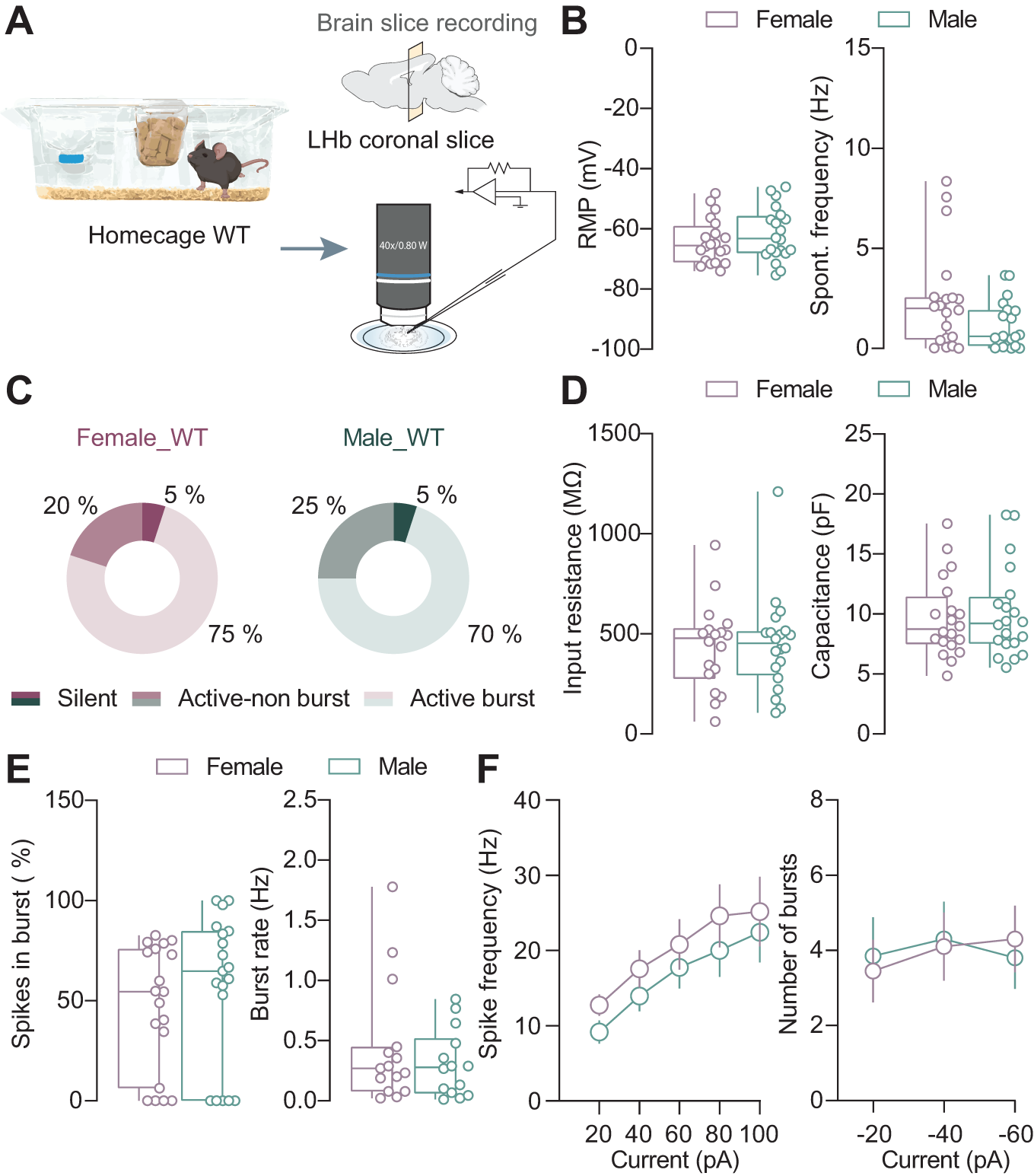

Supplement: Figure 2-2 — Ex vivo assessment of LHb neuronal activity in home-cage wild type female and male mice (A) Experimental timeline. All mice were sacrificed, and brains were sliced into coronal sections directly after being taken out of their home-cage. WT females: N= 2 mice. WT males: N= 2 mice. (B) Boxplots of RMP (left) (WT females vs WT males; -64.17 ± 1.664 mV vs -61.77 ± 1.955 mV; t = 0.933, df = 38, p = 0.357, unpaired t-test) and spontaneous action potential frequency (right) (WT females vs WT males; 2.363 ± 0.560 Hz vs 1.139 ± 0.266 Hz; U = 144.5, p = 0.136, Mann-Whitney test). WT females: n= 20 cells. WT males: n= 20 cells. (C) Pie chart for distribution of firing patterns in naïve female and male mice, numbers representing the percentage of each firing pattern (WT females vs WT males; X2 = 1.146, df = 2, p = 0.930, Chi-Square test). WT females: n= 20 cells. WT males: n= 20 cells. (D) Boxplot of input resistance (WT females vs WT males; 434.3 ± 50.83 MΩ vs 446.3 ± 53.47 MΩ; U = 179, p = 0.989, Mann-Whitney test). WT females: n= 18 cells. WT males: n= 20 cells. Boxplot of capacitance (WT females vs WT males; 9.643 ± 0.734 pF vs 10.040 ± 0.840 pF; U = 192.5, p = 0.846, Mann-Whitney test). WT females: n= 20 cells. WT males: n= 20 cells. (E) Boxplot of spikes in burst % (WT females vs WT males; 46.40 ± 7.193 % vs 56.09 ± 8.525 %, U = 143, p = 0.277, Mann-Whitney test). WT females: n= 19 cells. WT males: n= 19 cells (left). (Right) burst rate (WT females vs WT males; 0.441 ± 0.130 Hz vs 0.308 ± 0.075 Hz, U = 94, p = 0.644, Mann-Whitney test), WT females: n= 15 cells. WT males: n= 14 cells. (F) Plot of action potential frequency in response to different injected currents (left). (p = 0.986, F(4,152) = 0.090, Two-way ANOVA RM). Plot reporting number of bursts after hyperpolarization in response to different injected currents (right) (p = 0.441, F(2,76) = 0.828, Two-way ANOVA RM). WT females: n= 20 cells. WT males: n= 20 cells. Download Figure 2-2, TIF file. [file eneuro-12-ENEURO.0414-24.2025-s003.tif]

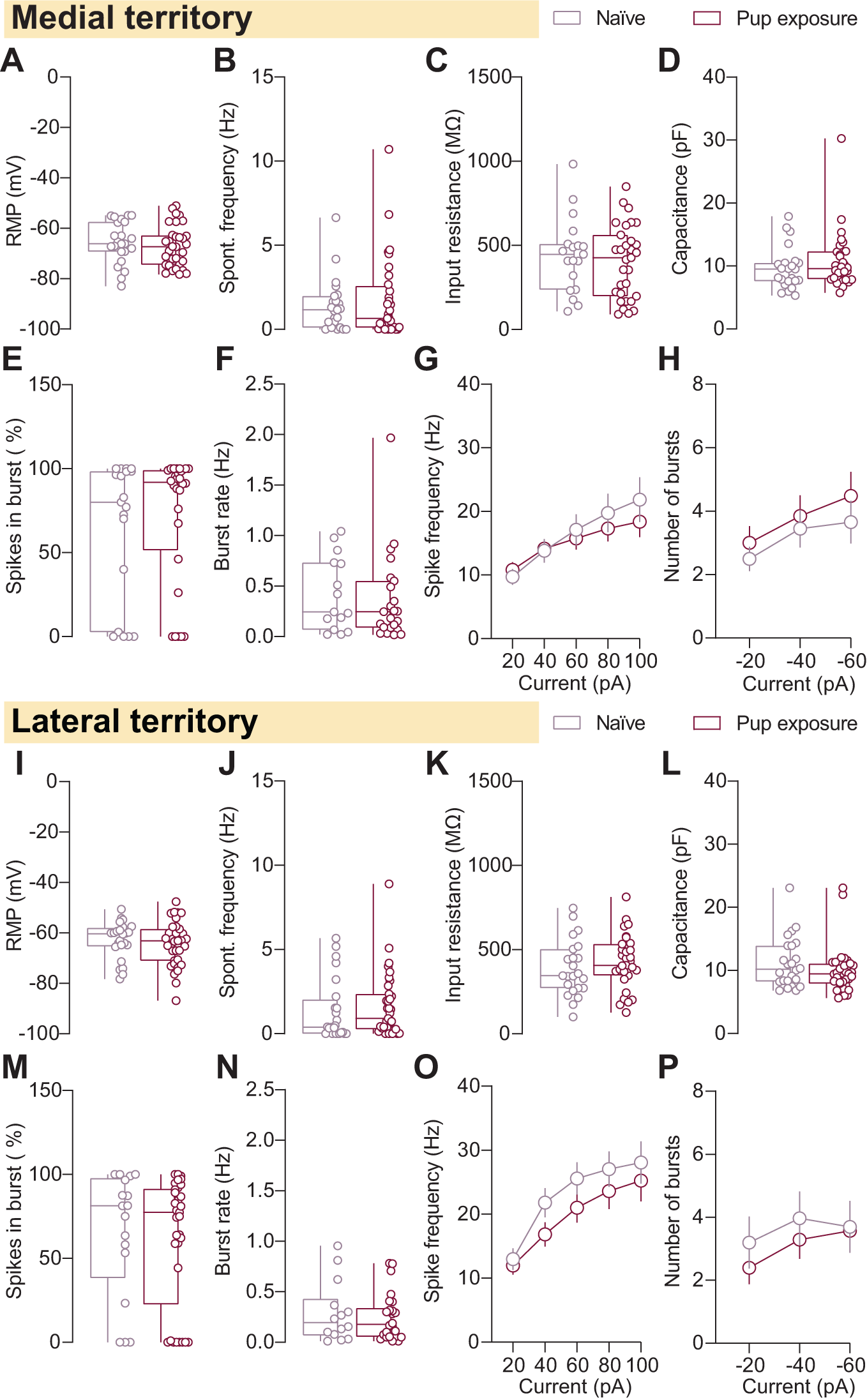

Supplement: Figure 4-1 — Lateral habenula territories and neuronal properties of naïve and pup-exposed female mice Data were analyzed based on the separation indicated in figure 4B. Naïve group: N= 6 mice. Pup exposure group: N= 8 mice. (A-H): medial territory. (A) Boxplot of RMP (naïve vs pup exposure; -65.61 ± 1.724 mV vs -67.07 ± 1.448 mV, t = 0.652, df = 53, p = 0.517, unpaired t-test). Naïve group: n= 23 cells. Pup exposure group: n= 32 cells. (B) Boxplot of spontaneous action potential frequency (naïve vs pup exposure; 1.407 ± 0.326 Hz vs 1.732 ± 0.426 Hz, U = 360.5, p = 0.902, Mann-Whitney test). Naïve group: n= 23 cells. Pup exposure group: n= 32 cells. (C) Boxplot of input resistance (naïve vs pup exposure; 440.8 ± 50.54 MΩ vs 402.2 ± 38.65 MΩ; t = 0.610, df = 48, p = 0.545, unpaired t-test). Naïve group: n= 19 cells. Pup exposure group: n= 31 cells. (D) Boxplot of capacitance (naïve vs pup exposure; 9.619 ± 0.698 pF vs 10.76 ± 0.787 pF; U = 309, p = 0.319, Mann-Whitney test). Naïve group: n= 23 cells. Pup exposure group: n= 32 cells. (E) Boxplot of spikes in burst % (naïve vs pup exposure; 63.91 ± 9.553 % vs 72.64 ± 7.249 %, U = 238, p = 0.548, Mann-Whitney test). Naïve group: n= 19 cells. Pup exposure group: n= 28 cells. (F) Boxplot of burst rate (naïve vs pup exposure; 0.417 ± 0.094 Hz vs 0.369 ± 0.092 Hz, U = 155.5, p = 0.621, Mann-Whitney test). Naïve group: n= 15 cells. Pup exposure group: n= 23 cells. (G) Plot of action potential frequency in response to different injected currents (p = 0.200, F(4,212) = 1.510, Two-way ANOVA RM). Naïve group: n= 23 cells. Pup exposure group: n= 32 cells. (H) Plot reporting number of bursts after hyperpolarization in response to different injected currents (p = 0.765, F(2,106) = 0.268, Two-way ANOVA RM). Naïve group: n= 23 cells. Pup exposure group: n= 32 cells. (I-P): lateral territory. (I) Boxplot of RMP (naïve vs pup exposure; -62.93 ± 1.500 mV vs -64.31 ± 1.480 mV; t = 0.632, df = 57, p = 0.530, unpaired t-test). Naïve group: n= 24 cells. [file eneuro-12-ENEURO.0414-24.2025-s004.tif]

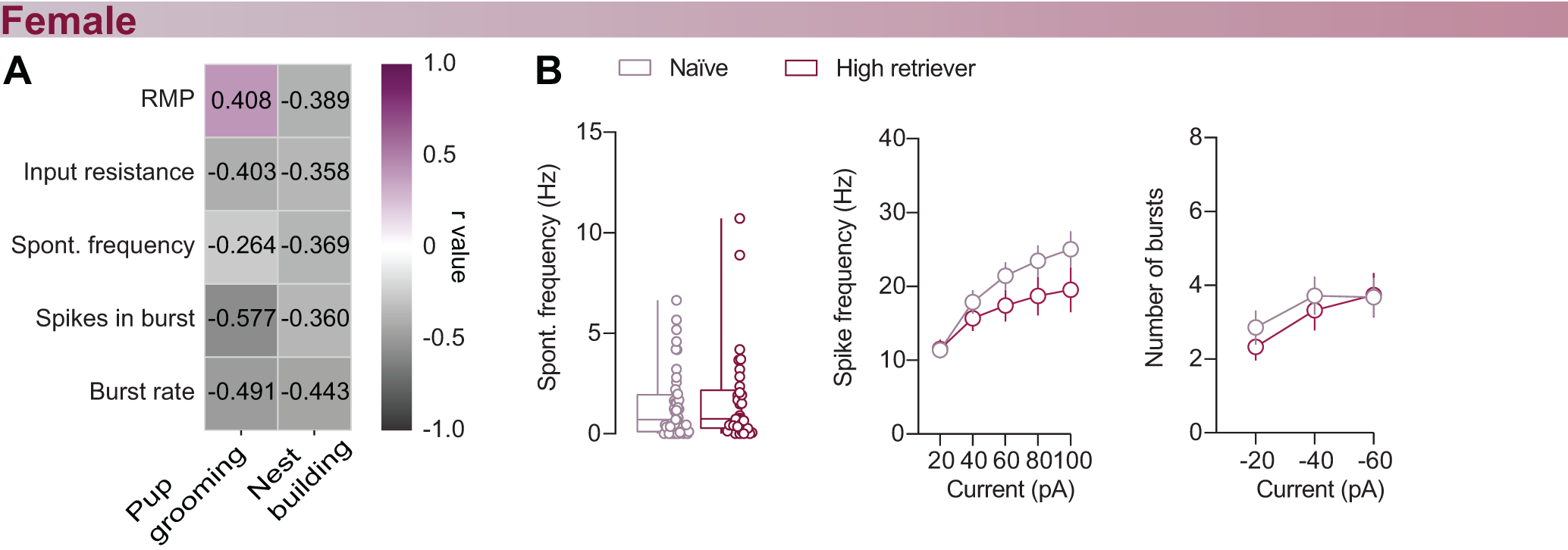

Supplement: Figure 4-2 — Correlation between behaviors and electrophysiological data in female mice (A) A correlation matrix illustrating the relationship between behaviors—pup grooming and nest building—and electrophysiological parameters: resting membrane potential (RMP), input resistance, spontaneous firing rate, spikes in bursts, and burst rate. The analysis involves the average electrophysiological data from each mouse and its behavioral time allocation, aggregated across a total of 8 females exposed to pups. Pearson correlation r value displayed. (B) Ex vivo data comparison between naïve females and females exhibiting high retrieval performance, naïve females: N= 6 mice, n= 47 cells, high retrieval: N=4 mice, n= 33 cells. Boxplot of spontaneous action potential frequency (naïve vs high retrieval; 1.375 ± 0.246 Hz vs 1.721 ± 0.421 Hz; U = 703, p = 0.481, Mann-Whitney test). Plot of action potential frequency in response to different injected currents (p = 0.077, F(4,312) = 2.132, Two-way ANOVA RM). Plot reporting number of bursts after hyperpolarization in response to different injected currents (p = 0.489, F(2,156) = 0.719, Two-way ANOVA RM). Download Figure 4-2, TIF file. [file eneuro-12-ENEURO.0414-24.2025-s005.tif]

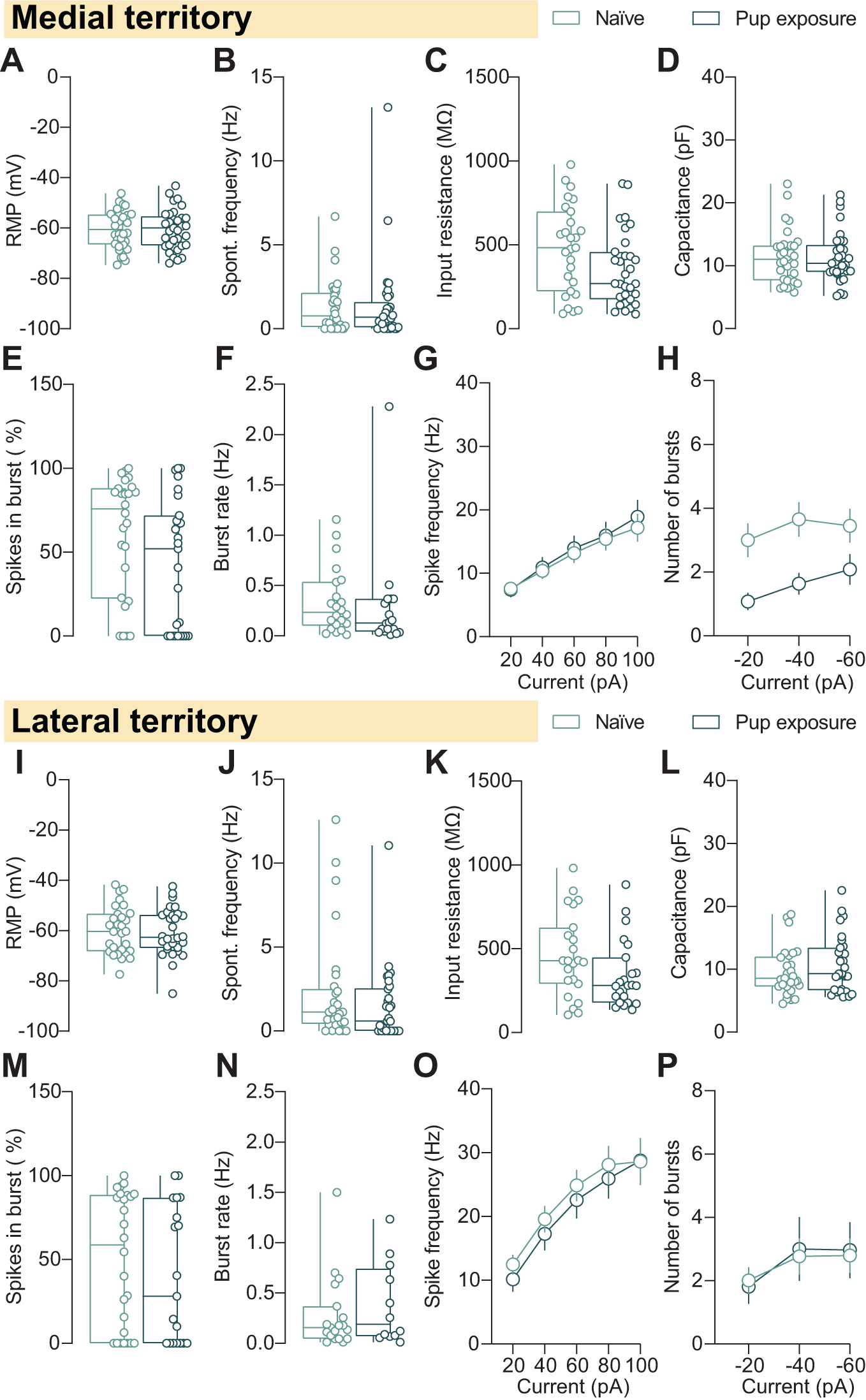

Supplement: Figure 4-3 — Lateral habenula territories and neuronal properties of naïve and pup exposure male mice Data were analyzed based on the separation indicated in figure 4G. Naïve group: N= 8 mice. Pup exposure group: N= 8 mice. (A-H): medial territory. (A) Boxplot of RMP (naïve vs pup exposure; -60.47 ± 1.342 mV vs -60.48 ± 1.389 mV, t = 0.005, df = 63, p = 0.996, unpaired t-test). Naïve group: n= 32 cells. Pup exposure group: n= 33 cells. (B) Boxplot of spontaneous action potential frequency (naïve vs pup exposure; 1.313 ± 0.276 Hz vs 1.350 ± 0.432 Hz, U = 488.5, p = 0.608, Mann-Whitney test). Naïve group: n= 32 cells. Pup exposure group: n= 33 cells. (C) Boxplot of input resistance (naïve vs pup exposure; 479.5 ± 50.19 MΩ vs 352.5 ± 39.17 MΩ; U = 298, p = 0.061, Mann-Whitney test). Naïve group: n= 27 cells. Pup exposure group: n= 31 cells. (D) Boxplot of capacitance (naïve vs pup exposure; 11.32 ± 0.749 pF vs 11.29 ± 0.695 pF; U = 526, p = 0.982, Mann-Whitney test). Naïve group: n= 32 cells. Pup exposure group: n= 33 cells. (E) Boxplot of spikes in burst % (naïve vs pup exposure; 60.69 ± 6.972 % vs 42.72 ± 7.447 %, U = 256, p = 0.090, Mann-Whitney test). Naïve group: n= 26 cells. Pup exposure group: n= 27 cells. (F) Boxplot of burst rate (naïve vs pup exposure; 0.341 ± 0.070 Hz vs 0.287 ± 0.122 Hz, U = 152, p = 0.216, Mann-Whitney test). Naïve group: n= 22 cells. Pup exposure group: n= 18 cells. (G) Plot of action potential frequency in response to different injected currents (p = 0.935, F(4,252) = 0.206, Two-way ANOVA RM). Naïve group: n= 32 cells. Pup exposure group: n= 33 cells. (H) Plot reporting number of bursts after hyperpolarization in response to different injected currents (p = 0.120, F(2,126) = 2.157, Two-way ANOVA RM). Naïve group: n= 32 cells. Pup exposure group: n= 33 cells. (I-P): lateral territory. (I) Boxplot of RMP (naïve vs pup exposure; -59.80 ± 1.756 mV vs -60.55 ± 1.711 mV; t = 0.306, df = 57, p = 0.761, unpaired t-test). Naïve group: n= 29 cells. Pup exposur [file eneuro-12-ENEURO.0414-24.2025-s006.tif]

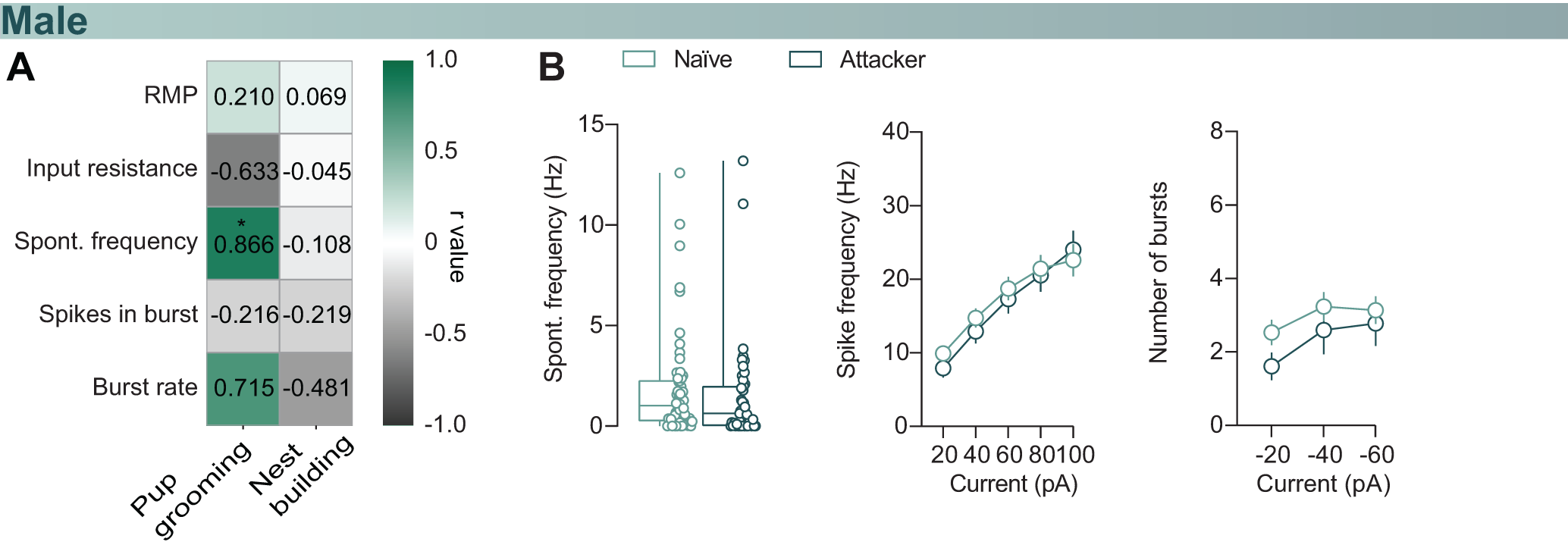

Supplement: Figure 4-4 — Correlation between behaviors and electrophysiological data in male mice (A) A correlation matrix illustrating the relationship between behaviors—pup grooming and nest building—and electrophysiological parameters: resting membrane potential (RMP), input resistance, spontaneous firing rate, spikes in bursts, and burst rate. The analysis involves the average electrophysiological data from each mouse and its behavioral time allocation, aggregated across a total of 8 males exposed to pups. One male was excluded due to immediately aggression toward pups right after experiment started. Pearson correlation r value displayed. (grooming the pup vs spontaneous firing rate: *p = 0.012) (B) Ex vivo data comparison between naïve males and males exhibiting attack behavior toward newborn. Naïve males: N= 8 mice, n= 61 cells, attacker: N=6 mice, n= 47 cells. Boxplot of spontaneous action potential frequency (naïve vs attacker; 1.779 ± 0.323 Hz vs 1.462 ± 0.372 Hz; U = 1216, p = 0.177, Mann-Whitney test). Plots of action potential frequency in response to different injected currents (p = 0.393, F(4,424) = 1.027, Two-way ANOVA RM). Plot reporting number of bursts after hyperpolarization in response to different injected currents (p = 0.354, F(2,212) = 1.044, Two-way ANOVA RM). Download Figure 4-4, TIF file. [file eneuro-12-ENEURO.0414-24.2025-s007.tif]
